# *Bacillus subtilis* EGY1 glucansucrase: optimization, characterization and immobilization using activated carrier of pectin-egg white protein beads

**DOI:** 10.1007/s13205-025-04609-7

**Published:** 2025-12-08

**Authors:** Shaymaa A. Ismail, Marwa I. Wahba, Shaimaa A. Nour, Amira A. Gamal, Asmaa Ezzat, Amal M. Hashem

**Affiliations:** 1 Chemistry of Natural and Microbial Products Department, Pharmaceutical and Drug Industries Research Institute, National Research Centre, 33 El Bohouth St., Dokki, P.O.12622, Giza, Egypt; 2 Centre of Scientific Excellence-Group of Advanced Materials and Nanotechnology, National Research Centre, 33 El Bohouth St., Dokki, P.O.12622, Giza, Egypt

**Keywords:** *Bacillus* subtilis, Glucansucrase, Optimization, Immobilization

## Abstract

Glucansucrases are the key biocatalysts in the industrial production of glucans. This study initially investigated glucansucrase production using the honey isolate *Bacillus subtilis* EGY1 followed by optimizing the enzyme productivity in which the optimized activity was 15-fold higher than the estimated initial activity. Moreover, the enzyme was immobilized using a carrier matrix of egg white protein (EWP) incorporated into pectin beads, activated with polyethyleneimine (PEI) and glutaraldehyde (GA). The optimal conditions for egg white protein and PEI concentrations, as well as pH, were determined using Box-Behnken design in which the estimated optimal conditions were EWP concentration of 1% and PEI processing conditions of 2.5% PEI concentration and 9.4 pH. At these optimal conditions, the immobilized enzyme exhibited high immobilization yield (93.87%) and efficiency (94.95%). Surface morphology, structural elements, and functional groups were analyzed using scanning electron microscopy (SEM), energy-dispersive X-ray spectroscopy, and Fourier-transform infrared spectroscopy. The immobilized enzyme demonstrated improved activity at alkaline pH (up to pH 9) and high temperatures (up to 75 °C), with reduced activation energy, approximately one-third that of the free enzyme. In addition, it retained > 50% activity after four reuse cycles.

**Supplementary Information:**

The online version contains supplementary material available at 10.1007/s13205-025-04609-7.

## Introduction

Glucansucrases, a class of glycosyltransferases that catalyze the production of oligosaccharides and polysaccharides by utilizing glucose from sucrose as a substrate (Monchois et al. 1999). These enzymes are predominantly found in lactic acid bacteria, including *Leuconostoc*, *Lactobacillus*, *Weissella*, and *Streptococcus* species (Monsan et al. 2000). Based on the type of the synthesized polysaccharides, glucansucrases are classified into dextransucrases, mutansucrases, alternansucrases, and reuteransucrases (Molina et al. 2021). The molecular structure, linkage types, and molar masses of the synthesized polysaccharides either dextran, mutan, alteran, or reuteran afford unique physicochemical properties, enabling their diverse applications in food and biomedical industries (Chen et al. 2021; Elgamily et al. 2019; Kavitake et al. 2024; Lin et al. 2024, Sharma and Jagadeesh Chandra Bose 2024).

As the demand for glucans increases, immobilization of glucansucrases has attracted the research focus due to its potential to enhance enzyme stability as well as providing reusability for continuous processing. Previous research has explored encapsulation for glucansucrase immobilization; however, challenges such as substrate and product diffusion, particle swelling, enzyme leakage, and impaired reusability has been estimated (Berensmeier et al. 2004; Reischwitz et al. 1995). Solid covalent enzyme immobilizers offer better stabilization under various conditions and significantly improve reusability (El-Shazly et al. 2024; Ismail et al. 2019; Rodrigues et al. 2021). However, previous studies have reported low immobilization yield and efficiency for glucansucrase using solid carriers (da Silva et al. 2019, 2020). Therefore, there is an importance for examining new combinations in the formation of solid carriers.

Proteins were formerly incorporated within covalent-immobilizers and their blending with polysaccharides promoted their activation process and provided finer covalent-immobilizers. Whey protein isolate, EWP, and soy protein isolate (SPI) were examples of proteins previously grafted onto variable polysaccharides during their activation into covalent-immobilizers (Wahba 2020a, 2022a). Given the possible benefits of blending proteins within polysaccharide-based immobilizers, the protein; EWP was blended with the polysaccharide; pectin in order to provide finer pectin covalent-immobilizer. Pectin is mainly obtained from the wastes of altered plants, such as citrus fruits and apples (Einhorn-Stoll et al. 2021). Pectin exhibits a backbone of galacturonic acid entities (Bellemjid et al. 2021). The anionic traits of these acid entities enabled the preparation of pectin covalent-immobilizers after activating calcium pectinate (CP) beads with the cationic PEI and GA. As regards to EWP, it is constituted from assorted proteins that exhibit much altered molecular weights and pIs. However, its major constituent (ovalbumin; 54% of EWP) offers a 4.5–4.7 pI (Lu et al. 2020; Razi et al. 2023). Accordingly, EWP would chiefly be anionic at pHs ≥ 8, which are the pHs commonly adopted during PEI grafting (Saleh et al. 2023; Wahba 2020b, 2021). The EWP anionic attributes would raise the anionic attributes of the CP beads. Accordingly, the CP-EWP—PEI ionic-interactions would be improved and finer pectin covalent-immobilizers would be prepared.

In this study, the honey isolate *Bacillus subtilis* EGY1 was estimated as a glucansucrase producer. The enzyme productivity was initially optimized using statistical experimental designs. Furthermore, the produced enzyme under the optimized conditions was precipitated by ammonium sulphate and immobilized using a newly constructed carrier in which EWP was blended with pectin. Moreover, Box-Behnken (BB) design was applied to identify the optimal EWP concentration, PEI concentration, and PEI pH that should be adopted for constructing the examined carrier.

## Materials and methods

### Microorganism

In the current study, *Bacillus subtilis* EGY1 (accession number PP038112), previously isolated from Saudi Arabia sider mountain honey samples at the National Research Centre^’^s Department of Chemistry of Natural and Microbial Products in Dokki, Giza, Egypt, was examined for enzyme production.

### Fermentation process and precipitation of the synthesized exo-polysaccharide

Fermentation was performed in 250 mL Erlenmeyer flasks containing 50 mL medium described previously by Hashem et al. (2018). The medium composition was sucrose (13.5 g%), wheat flour (2 g%), yeast extract (0.04 g%), K₂HPO₄ (0.4 g%), MgSO₄·7H₂O (0.01 g%), and ZnSO₄ (0.005 g%). It was inoculated by 6% v/v 24 h nutrient broth culture of the isolate and cultivated at 37 °C with shaking at 180 rpm for 24 h.

At the end of the fermentation period, the culture was centrifuged at 4000 rpm for 10 min at 4 °C. The resulting cell-free supernatant was subjected to ethanol precipitation (1:2 v/v) and air-dried in pre-weighed Petri dishes. The precipitated exopolysaccharide was hydrolyzed with 80% H₂SO₄, and the monosaccharide composition was analyzed via thin-layer chromatography (TLC) using acetonitrile:water (85:15 v/v) as the mobile phase and sulfuric acid-naphthol as the spraying reagent (Parlak et al. 2013).

Molecular weight determination of the precipitated polysaccharide was performed using a U-shaped Ubbelohde viscometer (Gaisford et al. 1986). Characterization of the polysaccharide was conducted using ^1^H and ^13^C nuclear magnetic resonance (NMR) and Fourier-transform infrared spectroscopy (FTIR).

### Enzyme quantification

The glucansucrase activity in the cell-free supernatant was quantified using a reaction mixture containing equal volumes of supernatant and 5% sucrose solution (prepared in 50 mM phosphate buffer, pH 6). The mixture was incubated at 50 °C for 30 min, and the reducing sugars were quantified using 3,5-dinitrosalicylic acid (DNS) method, with fructose as the standard (Miller 1959). One unit (U) of the enzymatic activity was defined as the amount of enzyme required to release 1 μmol of reducing sugars per minute. Protein concentration was determined using Lowry method with bovine serum albumin as the standard (Lowry et al. 1951).

### Statistical optimization of the enzyme productivity

Statistical optimization was conducted using Plackett–Burman (PB) and Box-Behnken (BB) designs. Seven independent variables were evaluated using the PB design: sucrose, urea, wheat flour, K₂HPO₄, MgSO₄·7H₂O, ZnSO₄ concentrations, and medium pH. Each variable was tested at low (−1) and high (+ 1) levels across eight experimental runs. The influence of each variable [E(Xi)] was determined using the following formula:

E(Xi) = 2(Σ Mi + − Mi −)/R, where Mi − and Mi + were enzyme activities at high and low variable levels, respectively, and R was the number of runs (Plackett and Burman 1946).

Significant variables (p ≤ 0.05) identified in the PB design were further optimized using the BB design. Each variable was examined at three levels (−1, 0, + 1) to identify the optimal conditions (Box and Behnken 1960).

### Immobilization of the produced enzyme

#### Preparation of CP-EWP/PEI/GA beads

Initially, a 6% pectin solution was mixed with an EWP suspension in a 1:1 ratio, resulting in a final concentration of 3% pectin and 1% EWP. The mixture was refrigerated overnight and then dripped into 5% CaCl_2_.2H_2_O solution using a syringe. The prepared CP-EWP beads were left in the solution for about 18 h, washed thoroughly, and activated sequentially with 3% PEI (pH 10.5) for 2 h and 5% GA for 1 h. Excess reagents were washed off after each activation step.

#### Loading process

The enzyme, precipitated with ammonium sulfate, was immobilized by incubating 1 mL of the enzyme solution (23.6 U/mg protein) with 1 g of activated beads overnight under shaking. After immobilization, washing of the beads was carried twice and the activity was inspected as described previously by suspending of the beads in 50 mM phosphate buffer pH 6. The immobilization yield (IY) and immobilization efficiency (IE) were calculated as follows:

$${\text{IY }}\left( \% \right)\, = \,\left( {{\mathrm{a}} - {\mathrm{b}}} \right)/{\mathrm{a}})*{1}00$$

$${\text{IE }}\left( \% \right)\, = \,\left( {{\mathrm{c}}/{\mathrm{a}} - {\mathrm{b}}} \right)*{1}00$$

where a, b and c were the loaded, un-bound and the bounded enzyme activities (U/g), respectively.

#### Optimizing the CP-EWP/PEI/GA beads

The optimization of CP-EWP/PEI/GA beads for enzyme immobilization was conducted using BB design in which the examined variables were the concentration of EWP (A), the concentration of PEI (B), and PEI pH (C). During the BB design, the prepared 6% pectin solution was blended with either distilled water or EWP suspension in order to attain final EWP concentrations of 0, 1, and 2%. After that, the beads were processed as described in Sect. “Preparation of CP-EWP/PEI/GA beads”, with PEI concentrations set at 2.5%, 3.5%, and 4.5%, and PEI pH adjusted to 9.4, 10.5, and 11.6.

#### Activation and immobilization processes analysis

##### Scan electron microscope (SEM)

After drying the samples, the surface morphology of CP-EWP and CP-EWP/PEI/GA beads was analyzed using SEM Quanta 250 FEG, Netherlands.

##### Elemental analysis

The elemental composition of CP-EWP, CP-EWP/PEI, CP-EWP/PEI/GA, and enzyme-loaded CP-EWP/PEI/GA beads was analyzed using an Octane Pro EDAX (AMETEK, USA). Carbon, nitrogen, and oxygen contents were quantified for all samples. Nonetheless, calcium was determined in the CP-EWP beads; thus, it^’^s percent was presented. Moreover, sulfur was detected in the enzyme loaded beads; thus, it^’^s percent was presented.

##### Fourier transform infrared spectroscopy scrutinization

FTIR spectra of CP-EWP, CP-EWP/PEI, CP-EWP/PEI/GA, and enzyme-loaded CP-EWP/PEI/GA beads were recorded in the 4000–400 cm⁻^1^ range to identify functional groups and confirm structural modifications during the immobilization process.

#### Inspection of the physical stability of the CP-EWP/PEI/GA immobilizers

##### Mechanical stability

A 5 mL glass vial, which contained an accurately weighed amount of the CP-EWP/PEI/GA beads (0.5 g), 1 g glass beads (1.05 mm diameter), and 1 mL distilled water, was vortexed for 3 min and 20 s. Such vortexing would fragment the CP-EWP/PEI/GA beads if they were mechanically unstable. Afterwards, the vial contents were sieved through a standard sieve (No. 16, aperture 1.18 mm) in order to eliminate any minute beads fragments. Finally, the CP-EWP/PEI/GA beads, which were kept by the sieve, were weighed and their weight was presented as a percent of the initial beads weight (0.5 g) (Wahba 2016).

##### Stability within aqueous media

The CP-EWP/PEI/GA beads (0.15 g) were accurately weighed, and then they were immersed in either 3 mL distilled water or 3 mL 0.05 M acetate buffer pH 5. After specified periods, the beads were removed from the aqueous media, padded between two filter-papers to eliminate excess moisture, and weighed. This weight was presented as a percent of the initial beads weight (0.15 g).

#### Immobilized enzyme characterization

##### pH effect

Different buffer solutions (50 mM); acetate buffer (pH 4–5), phosphate buffer (pH 6–8), and Tris/HCl buffer (pH 9) were used to determine the pH influence on the immobilized enzyme activity in comparison to the free form at various pH values.

##### Temperature effect

The temperature influence on the enzyme activity was assessed at 30 to 75 °C range of temperatures. The reported formula was used to determine the enzyme activation energy (Ea) from the Arrhenius plot in which ln (relative activity) was graphed versus 1/temperature (Kelvin).

Slope = −Ea/R, where R is the gas constant.

Furthermore, the thermal stability of the enzyme was assessed by estimating the residual enzymatic activity following pre-incubation of the enzyme in absence of its substrate for durations up to 120 min, at 45 to 60 °C temperature range. The preliminary activity (without pre-incubation) was the 100%. Following that, the enzyme^’^s thermostability parameters of the enzyme were procured as follows:

$${\mathrm{T}}_{{{1}/{2}}} \, = \,{\mathrm{ln}}\left( {2} \right)/{\mathrm{Kd}}$$

Decimal reduction time (*D*-value) = ln(10)/Kd, where, Kd was the thermal deactivation rate constant.

##### Substrate concentration effect

Under the optimal conditions, altered sucrose concentrations (10 to 100 mg mL^−1^) were adopted during the enzyme activity assessment.

#### Operational stability

The enzyme reusability was tested via determining its activity for several cycles in which the reaction solution after each cycle was subjected to analysis while the beads were thoroughly washed with distilled water before examining in the new reaction. Moreover, the immobilized enzyme storage stability was inspected weekly up to 4 weeks.

### Biological activities of the precipitated polysaccharide

#### Prebiotic activity

According to Azmi et al. (2012), the growth-stimulating potential of the investigated polysaccharides for three *Lactobacillus* probiotics; *L. casei, L. reuteri, and L. helveticus*, was assessed in relative to the pathogenic *Escherichia coli*. Ten milliliters of the media (MRS for probiotics and nutritional broth for the pathogen) supplemented with 30 mg of the samples under examination were inoculated with aliquots from each bacterial culture (0.1 ml). Following a 37 °C incubation for 24 h, the sample optical density (O.D.) was detected at 625 nm in comparison to an uninoculated media blank (Ismail and Emran 2020). By dividing the pathogenic culture O.D. by the probiotic bacterial culture O.D., the prebiotic index (I) was determined.

#### Antimicrobial activity

The well diffusion method (Mishra and Prasad 2005) was adopted while assessing the antimicrobial activity versus *Candida albicans* ATCC 10231, *Escherichia coli* ATCC 8739, and *Staphylococcus aureus* ATCC 6538. Using nutrient agar sterile petri dishes, each strain (0.2 mL of cell suspension corresponding to a 0.5 McFarland standard solution) was evenly distributed throughout the medium^’^s surface to measure the antibacterial activity. The analyzed sample (50 mg) was added to each of the 7 mm-diameter wells created in the agar plates using a sterilized glass Pasteur pipette. Afterwards, the plates were put in 37 °C incubator for 24 h. The inhibition zone diameters (mm) were estimated in which the average of two distinct runs of each experiment were represented.

The antifungal activity against *Candida albicans* ATCC 10231 was conducted: a single pure colony of the strain was first cultured in 10 millilitres of Sabouraud dextrose broth. It was then put in 30 °C incubator for 24 h. Cultured broth (0.2 mL) was evenly spread across the top of Sabouraud dextrose agar petri plates via a sterile swap. The analysed sample (0.1 mL) was then added to wells created in the agar plates using sterile glass Pasteur pipettes, which had a diameter of 7 mm. The zones of inhibition (mm) on the plates were recorded following a 24 h incubation period at 30 °C.

#### Fibrinolytic and anticoagulant activity

The fibrinolytic activity was given as the percentage of the plasma clot that was lysed (Pharmacopoeia 1960). In clean glass test tubes, 0.9% (w/v) saline solution (0.8 ml), prepared plasma (1.0 ml), and 2% (w/v) calcium chloride (0.2 ml) were put and mixed. Afterwards, the tubes were submerged in a 37 °C water-bath. After clotting was accomplished, either the analyzed sample (2 mg/tube), standard Hemoclar (2 mg/tube), or saline solution (0.9% w/v; 1 ml) was added as the positive control or negative control. After 24 h at 37 °C, the plasma clot lysis percentage was noted and compared with standard Hemoclar (Pentosan sulfuric polyester, product of Clin Midy. Paris).

On the other hand, the anticoagulant activity of the examined samples in compare to standard sodium heparin solution (presenting 1 mg/tube) was evaluated. In separate tubes, 0.9% w/v saline solution as the negative control (0.8 ml), standard sodium heparin solution (presenting 1 mg/tube) as the positive control, or the examined sample (1 mg/tube) was placed then human plasma (1 ml) and 2% (w/v) calcium chloride (0.2 ml) were put. Time was promptly noted, each tube was sealed, and the contents were combined by inverting the tube three times until the whole inner surface was moist. The clotting time was then calculated and compared with standard heparin purchased from Sigma-Aldrich, St. Louis, MO, USA.

### Statistical analysis

The results were given as the average ± standard deviation for triplicate experimental runs with duplicate measurements of each replicate. Excel data analysis was used for regression analysis, and the BB design was analyzed by Design-Expert 13 statistical software.

## Results

### The applied *Bacillus subtilis* produced dextran polysaccharide with various molecular weight under submerged fermentation of sucrose

**Fig. 1:**
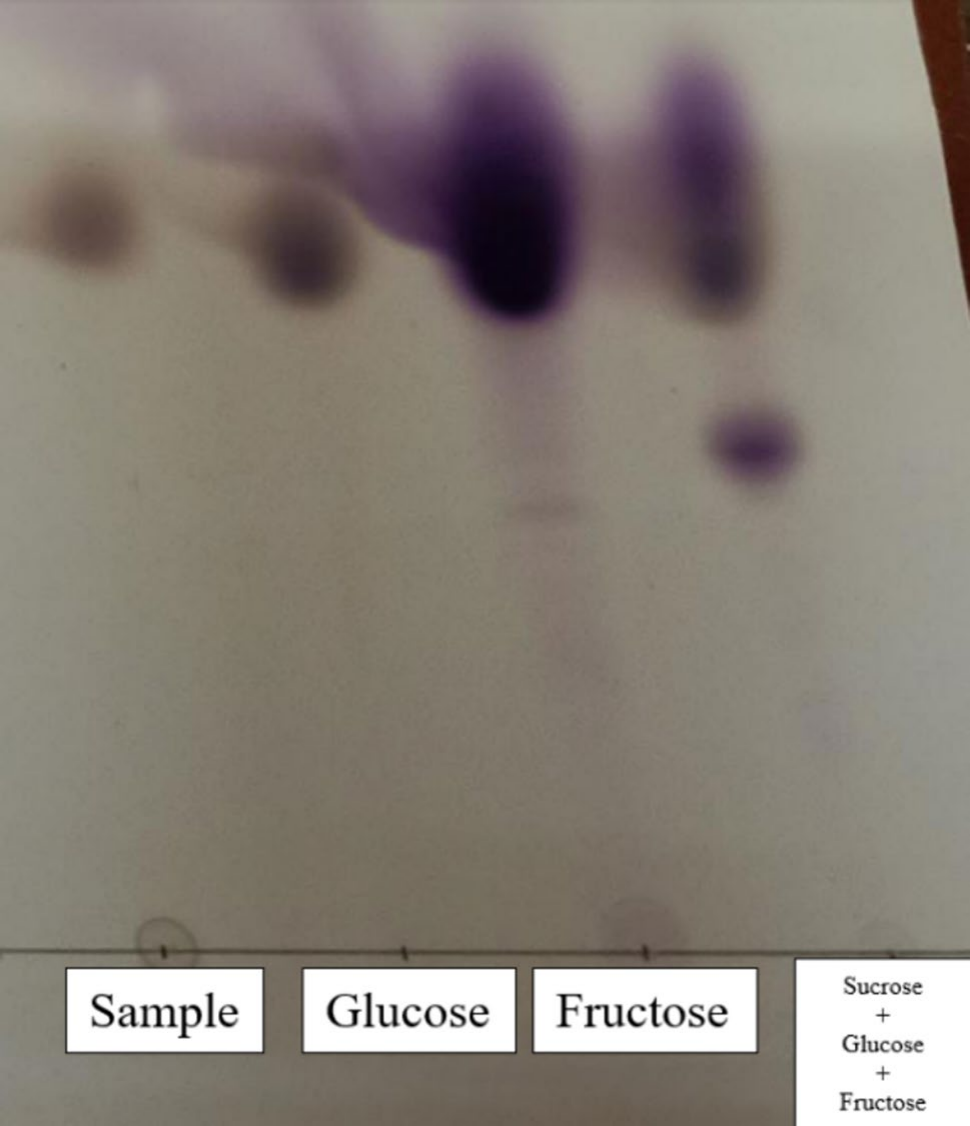
TLC analysis of the acid hydrolyzed precipitated exo-polysaccharide sample using glucose, fructose and sucrose as standards

Initially, the visual appearance of the ethanol precipitated cell-free culture indicated the formation of a gummy mass. After air-drying, the amount of the precipitate was estimated as 13.4 g/100mL and the TLC analysis (Fig. 1) of the acid-hydrolyzed sample indicated that glucose was the main component. Moreover, the molecular weight determination indicated that the precipitated polysaccharide produced under the fermentation conditions reported in Sect. “Fermentation process and precipitation of the synthesized exo-polysaccharide” was a complex glucan polymer with molecular weight of 75 KDa. By examining the impact of some cultural and nutritional conditions (incubation period, temperature, carbon and nitrogen content) on the molecular weight of the precipitated polysaccharide, a significant variation ranging from 51 to 125 KDa was observed by varying the sucrose concentration. Moreover, the highest estimated molecular weight was 168 KDa, estimated by the addition of wheat flour at concentration of 8% (Table 1).

**Table 1:** The effect of some cultural and nutritional parameters on the molecular weight of the precipitated polysaccharide as well as *Bacillus subtilis* EGY1 glucansucrase productivity

*Incubation period (h)*	6	10	18	24*	30	–		
Enzyme activity (U/mL)	2.201 ± 0.200	2.983 ± 0.010	3.402 ± 0.492	3.257 ± 1.295	3.031 ± 0.144			
Molecular weight of the precipitated EPS (KDa)	48.26 ± 0.12	61.29 ± 0.09	69.71 ± 0.05	75.84 ± 0.15	71.25 ± 0.18			
*Temperature (°C)*	25	28	30	33	37*	–		
Enzyme activity (U/mL)	9.319 ± 0.069	11.688 ± 0.170	5.674 ± 0.049	4.216 ± 0.028	3.432 ± 0.160			
Molecular weight of the precipitated EPS (KDa)	69.34 ± 0.20	75.84 ± 0.06	70.15 ± 0.14	69.96 ± 0.15	67.06 ± 0.07			
*Sucrose concentration (%)*	7.5	10	13.5*	20	25	30		–
Enzyme activity (U/mL)	9.361 ± 0.270	9.85 ± 0.413	11.682 ± 0.087	12.649 ± 0.028	8.018 ± 0.059	5.496 ± 0.035		
Molecular weight of the precipitated EPS (KDa)	51.12 ± 0.14	52.21 ± 0.05	75.84 ± 0.09	125.14 ± 0.17	100.99 ± 0.05	88.67 ± 0.04		
*Nitrogen source*	Yeast extract *	Peptone	(NH_4_)_2_SO_4_	Urea	Corn steep liquor	–		
Enzyme activity (U/mL)	12.615 ± 0.760	10.083 ± 0.722	10.097 ± 1.207	22.362 ± 0.638	7.760 ± 0.419			
Molecular weight of the precipitated EPS (KDa)	125.14 ± 0.19	76.26 ± 0.10	75.76 ± 0.02	130.81 ± 0.07	70.21 ± 0.15			
*Urea concentration (%)*	0.01	0.02*	0.04	0.06	0.08	0.1	–	
Enzyme activity (U/mL)	21.205 ± 1.609	22.362 ± 1.859	27.929 ± 1.526	31.160 ± 1.068	24.961 ± 0.957	20.734 ± 0.499		
Molecular weight of the precipitated EPS (KDa)	85.64 ± 0.05	100.81 ± 0.09	115.44 ± 0.03	142.93 ± 0.13	85.65 ± 0.15	70.66 ± 0.06		
*Wheat flour concentration (%)*	0.5	1	2*	4	6	8	10	
Enzyme activity (U/mL)	14.0566 ± 0.199	20.538 ± 3.068	29.742 ± 2.230	30.561 ± 3.129	32.174 ± 1.615	39.402 ± 1.595	38.692 ± 3.776	
Molecular weight of the precipitated EPS (KDa)	120.69 ± 0.04	127.96 ± 0.07	142.93 ± 0.17	153.18 ± 0.16	160.26 ± 0.09	168.26 ± 0.08	168.26 ± 0.03	

*Control

Polysaccharides of different molecular weight (51, 75 and 100 KDa) produced by the fermentation of different sucrose concentration (7.5, 13.5 and 25%) in addition to the one with the highest molecular weight (168 KDa) were named D1, D2, D3 and D4, respectively and characterized by FTIR and NMR analysis. The functional groups of the four precipitated polysaccharides were determined by FTIR analysis in which the characteristic polysaccharide profile was estimated; major peaks around 3290 and 2900 cm^−1^ corresponded to O–H and C–H stretching, peak around 1650 cm^−1^ corresponded to the presence of C–O group, peaks at about 1400 cm^−1^ corresponded to C–H, C–OH or C–O bending, the strongest peak in the region of 910–1170 cm − 1 corresponded to C–O–C and C–O stretching (Supplementary Fig. 1). Moreover, the NMR spectra provided strong evidence for the main structural features of dextran in which the anomeric proton peak at *δ* 4.86 ppm in the ^1^H NMR spectra indicated the domination of α-(1 → 6) glucosyl linkages (Supplementary Fig. 2).

### Different molecular weight dextrans possessed different biological activities

Following their characterization, the chosen polysaccharides with varying molecular weights were investigated as prebiotic, antibacterial, fibrinolytic, and anticoagulant agents. Comparing the growth densities of the pathogenic *Escherichia coli* and the probiotic bacteria *L. reuteri, L. helveticus,* and *L. casei* serves as the foundation for determining the prebiotic action. Only samples D2 and D4 exhibited prebiotic activities with *L. reuteri* and *L. helveticus*, but all samples showed prebiotic activity with *L. casei* (Table 2).

**Table 2:** Prebiotic activity

Sample	Prebiotic index (I)		
	*Lactobacillus casei*	*Lactobacillus reuteri*	*Lactobacillus helveticus*
D1	2.52 ± 0.06	0.46 ± 0.13	0.69 ± 0.27
D2	1.95 ± 0.08	1.36 ± 0.12	1.75 ± 0.04
D3	1.66 ± 0.15	0.87 ± 0.05	0.94 ± 0.14
D4	2.76 ± 0.18	1.89 ± 0.07	1.13 ± 0.09

I = ratio of bifidogenic bacteria growth/pathogenic *Escherichia coli* growth

I = negative effect when equal or less than 1

I = positive effect when more than 1

**Fig. 2:**
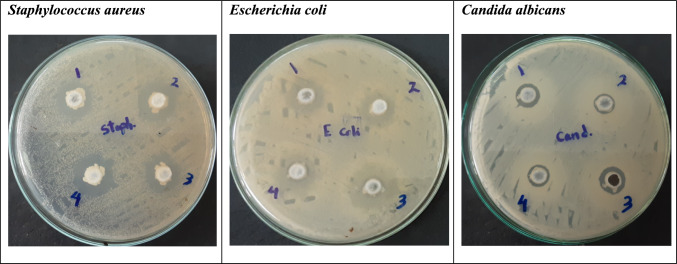
Antimicrobial activity of the precipitated exo-polysaccharide samples against *Staphylococcus aureus* ATCC 6538, *Escherichia coli* ATCC 8739, and *Candida albicans* ATCC 10231 in which 1, 2, 3 and 4 were the precipitated dextran^’^s of molecular weight 51, 75, 100 and 168 KDa, respectively

The antimicrobial activity indicated that all of the examined samples possessed varied activity against *Staphylococcus aureus* ATCC 6538 and *Candida albicans* ATCC 10231 without the estimation of any activity against *Escherichia coli* ATCC 8739 (Fig. 2, Table 3). Moreover, the results shown in Table 4 indicated that the precipitated dextrans, D3 and D4, possessed fibrinolytic and anticoagulant activity equivalent or higher to that of the commercial standards.

**Table 3:** MIC values

Sample	MIC value (mg/mL)	
	*Staphylococcus aureus* ATCC 6538	*Candida albicans* ATCC 10231
D1	62.5	250
D2	62.5	500
D3	62.5	125
D4	31.25	500

**Table 4:** Fibrinolytic and anticoagulant activities

Sample	Fibrinolytic activity (%)	Anticoagulant activity (Clotting time in sec)
D1	15 ± 0.17	18 ± 0.09
D2	50 ± 0.25	33 ± 0.18
D3	60 ± 0.06	145 ± 0.06
D4	40 ± 0.09	91 ± 0.04
Standard (Hemoclar)	40	–
Standard (Heparin)	–	90

### Glucansucrase was produced under the described fermentation conditions

The estimated glucansucrase activity produced herein by the fermentation of sucrose using the isolated *Bacillus subtilis* was 3.26 U/mL that increased to 38.4 U/mL by the use of sucrose of 20% (carbon source) and urea of 0.06 (nitrogen source) with the addition of wheat flour of 8% that incubated at 30 ºC for 18 h. In addition, by examining the impact of the fermentation process cultural and nutritional circumstances on the enzyme activity, a direct relationship between the enzyme activity and the molecular weight of the precipitated polysaccharide was determined (Table 1).

### *Bacillus subtilis* EGY1 glucansucrase was statistically optimized

**Fig. 3:**
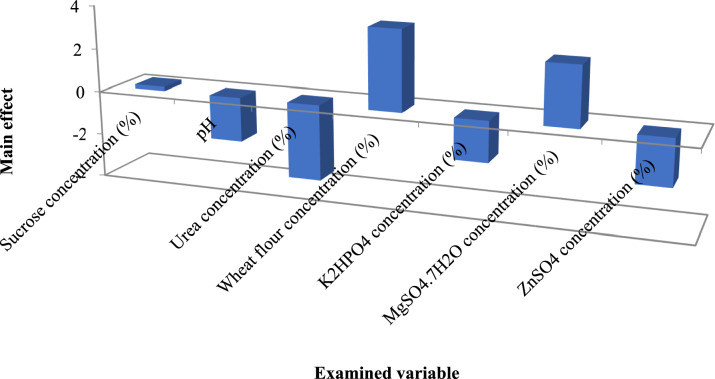
Main effect results of the examined variables on the enzyme productivity in Plackett–Burman design

**Fig. 4:**
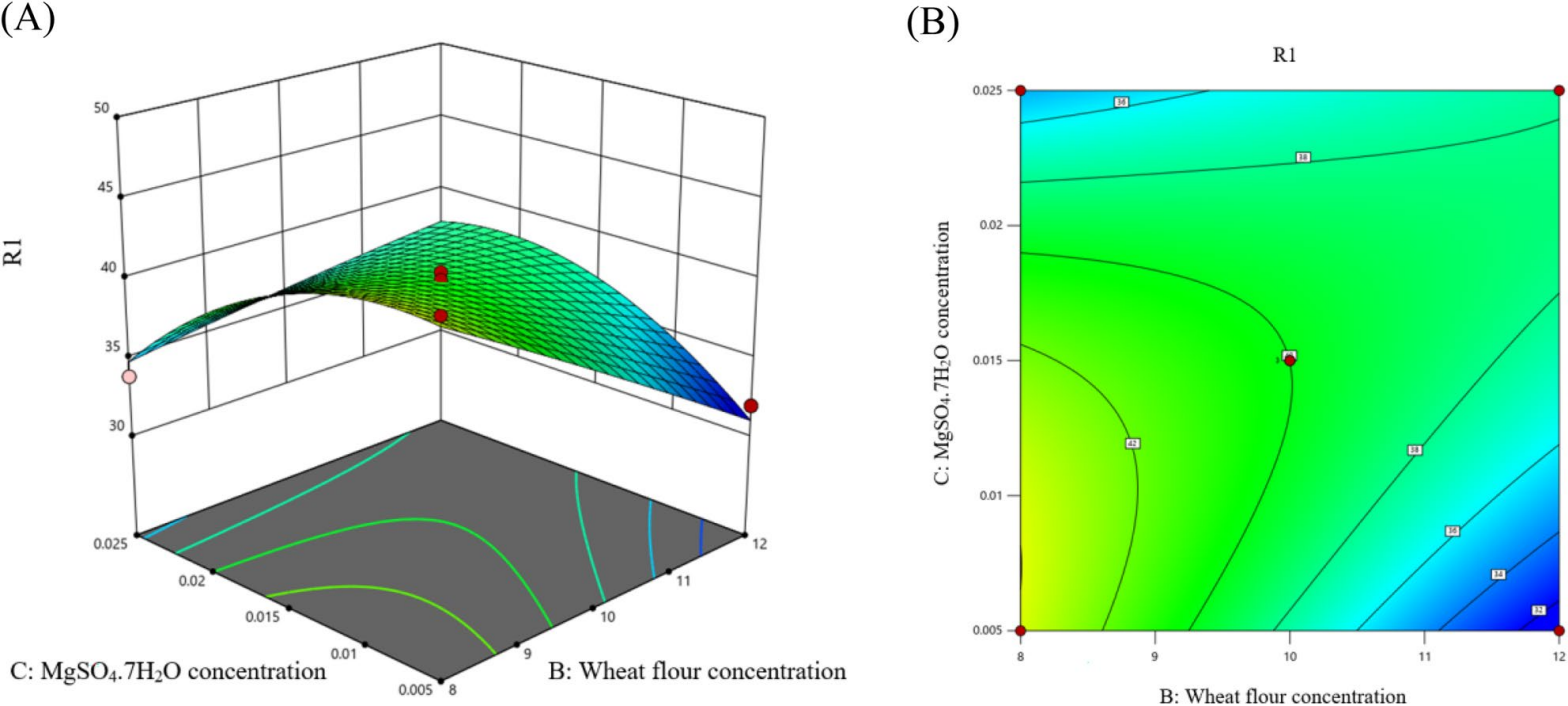
3D surface (**A**) and contour (**B**) plots monitoring the impact of the concentration of wheat flour and MgSO_4_.7H_2_O on the glucansucrase activity (R1) in which the third variable fixed at its central level

Initially, PB was applied to evaluate the impact of seven variables on the enzyme productivity at −1 and + 1 values as illustrated in Table 5. Multiple regression analysis of the results indicated that the coefficient of determination (R^2^) value for the applied design was 0.859 and the ANOVA analysis indicated that the F-value was 6.969 with P-value of 0.007, suggesting that the differences in the experimental results were unlikely to be due to chance. Moreover, the analysis indicated the significant impact of the concentration of urea, wheat flour and MgSO_4_.7H_2_O while the impact of the other 4 variables was insignificant (Supplementary Table 1). The insignificant variables were adjusted on their −1 values in the second step of optimization. On the other hand, the significant variables were adjusted on the base of their exerted main effect shown in Fig. 3 in which the concentration of urea possessed a negative effect while the concentration of wheat flour and MgSO_4_.7H_2_O possessed positive effect. The negative effect estimated that the higher effect of the variable achieved at −1 value while the positive effect was at + 1 value. These significant variables were furtherly optimized by applying BB design (Table 6). The analysis of the results indicated that the R^2^ value for the applied design was 0.9624 and the F-value was 14.24 with *P*-value of 0.005. Moreover, the analysis indicated the significance of the linear (A, B) and the quadratic (A^2^, C^2^) model terms in addition to the interactive term between the concentration of wheat flour and MgSO_4_.7H_2_O (BC) that was clearly estimated by the steeply curves shown in Fig. 4. In addition, the analysis indicated the insignificance of the lack of Fit (Supplementary Table 2).

$$\begin{aligned}{\text{Glucansucraseactivity }} & = { 4}0 \, {-}{ 1}.{\text{32A }}{-}{ 2}.{\text{47B }}{-} \, 0.{\text{5735C }} + \, 0.{8}0{\text{63AB }}\\ & \quad {-}{ 1}.{\text{4AC }} + { 3}.{\text{96BC }} + { 3}.{\mathrm{96A}}^{{2}} {-} \, 0.{\text{1858 B}}^{{2}} {-}{ 2}.{\text{96 C}}^{{2}} \end{aligned}$$

**Table 5:** Plackett–Burman design

Run number	Sucrose concentration (%)	pH	Urea concentration (%)	Wheat flour concentration (%)	K_2_HPO_4_ concentration (%)	MgSO_4_.7H_2_O concentration (%)	ZnSO_4_ concentration (%)	Enzyme activity (U/mL)
1	15 (−)	7.5 (−)	0.04 (−)	10 (+)	0.5 (+)	0.015 (+)	0.0025 (−)	40.683
2	20 (+)	7.5 (−)	0.04 (−)	6 (−)	0.3 (−)	0.015 (+)	0.0075 (+)	36.973
3	15 (−)	9.5 (+)	0.04 (−)	6 (−)	0.5 (+)	0.005 (−)	0.0075 (+)	30.171
4	20 (+)	9.5 (+)	0.04 (−)	10 (+)	0.3 (−)	0.005 (−)	0.0025 (−)	38.008
5	15 (−)	7.5 (−)	0.08 (+)	10 (+)	0.3 (−)	0.005 (−)	0.0075 (+)	34.254
6	20 (+)	7.5 (−)	0.08 (+)	6 (−)	0.5 (+)	0.005 (−)	0.0025 (−)	31.044
7	15 (−)	9.5 (+)	0.08 (+)	6 (−)	0.3 (−)	0.015 (+)	0.0025 (−)	33.366
8	20 (+)	9.5 (+)	0.08 (+)	10 (+)	0.5 (+)	0.015 (+)	0.0075 (+)	33.263

**Table 6:** Box-Behnken design

Run number	Independent variables			Enzyme activity (U/mL)
	A Urea concentration (%)	B Wheat flour concentration (%)	C MgSO_4_.7H_2_O concentration (%)	
1	−(0.02)	−(8)	0 (0.015)	48.60274
2	+ (0.06)	−(8)	0 (0.015)	43.18145
3	−(0.02)	+ (12)	0 (0.015)	41.74706
4	+ (0.06)	+ (12)	0 (0.015)	39.49616
5	−(0.02)	0 (10)	−(0.005)	39.60159
6	+ (0.06)	0 (10)	−(0.005)	41.01637
7	−(0.02)	0 (10)	+ (0.025)	42.81121
8	+ (0.06)	0 (10)	+ (0.025)	38.611
9	0 (0.04)	−(8)	−(0.005)	44.45647
10	0 (0.04)	+ (12)	−(0.005)	31.93922
11	0 (0.04)	−(8)	+ (0.025)	33.83704
12	0 (0.04)	+ (12)	+ (0.025)	37.1668
13	0 (0.04)	0 (10)	0 (0.015)	40.49656
14	0 (0.04)	0 (10)	0 (0.015)	39.49616
15	0 (0.04)	0 (10)	0 (0.015)	39.99636

Validation of the model was carried out by applying the predicted optimized conditions (0.02% urea concentration, 8.001% wheat flour concentration and 0.01% MgSO_4_.7H_2_O concentration with the highest desirability value that led to the production of 49.485 U/mL that was close to the predicted optimal activity (48.696 U/mL).

### Optimizing the activation of the CP-EWP beads in order to efficiently immobilize the produced glucansucrase

The prepared CP-EWP/PEI/GA beads were examined for the immobilization of the precipitated enzyme (70% ammonium sulphate precipitated fraction with recovery percentage of 11.46% that possessed 23.57 U/mg protein specific activity and 6.39 purification fold). BB design was employed to disclose the optimal EWP concentration, which should be blended within the CP beads to maximize the enzyme IE. Moreover, the PEI optimal concentration and pH were also inspected via the applied design as the anionic attributes of EWP would directly influence the CP-EWP/PEI interactions (Table 7). The quadratic model was adequate to interpret the applied design.

$$\begin{aligned} {\text{IE }} &= { 82}.{33 } + { 5}.{\text{43 A }} - { 6}.{\text{11 B }} - { 14}.{\text{17 C }} + \, 0.{9}0{\text{ AB }} \\ & \quad - { 3}.{\text{16 AC }} + \, 0.{\text{97 BC }} - { 2}0.{6}0{\text{ A}}^{{2}} - { 5}.{2}0{\text{ B}}^{{2}} - { 2}.{\text{48 C}}^{{2}} \end{aligned}$$

**Table 7:** BB design for optimizing the immobilizers construction

Run	A: EWP concentration (%, w/w)	B: PEI concentration (%, w/w)	C: PEI pH	IE (%)
1	2 (+ 1)	3.5 (0)	9.4 (−1)	81.11
2	1 (0)	4.5 (+ 1)	11.6 (+ 1)	56.29
3	1 (0)	3.5 (0)	10.5 (0)	69.63
4	1 (0)	3.5 (0)	10.5 (0)	84.80
5	1 (0)	3.5 (0)	10.5 (0)	83.77
6	1 (0)	2.5 (−1)	9.4 (−1)	94.95
7	2 (+ 1)	4.5 (+ 1)	10.5 (0)	49.27
8	2 (+ 1)	2.5 (−1)	10.5 (0)	69.02
9	0 (−1)	3.5 (0)	9.4 (−1)	58.33
10	0 (−1)	2.5 (−1)	10.5 (0)	65.57
11	2 (+ 1)	3.5 (0)	11.6 (+ 1)	53.86
12	1 (0)	3.5 (0)	10.5 (0)	86.44
13	0 (−1)	4.5 (+ 1)	10.5 (0)	42.24
14	1 (0)	4.5 (+ 1)	9.4 (−1)	90.10
15	1 (0)	2.5 (−1)	11.6 (+ 1)	57.25
16	0 (−1)	3.5 (0)	11.6 (+ 1)	43.71
17	1 (0)	3.5 (0)	10.5 (0)	87.01

**Fig. 5:**
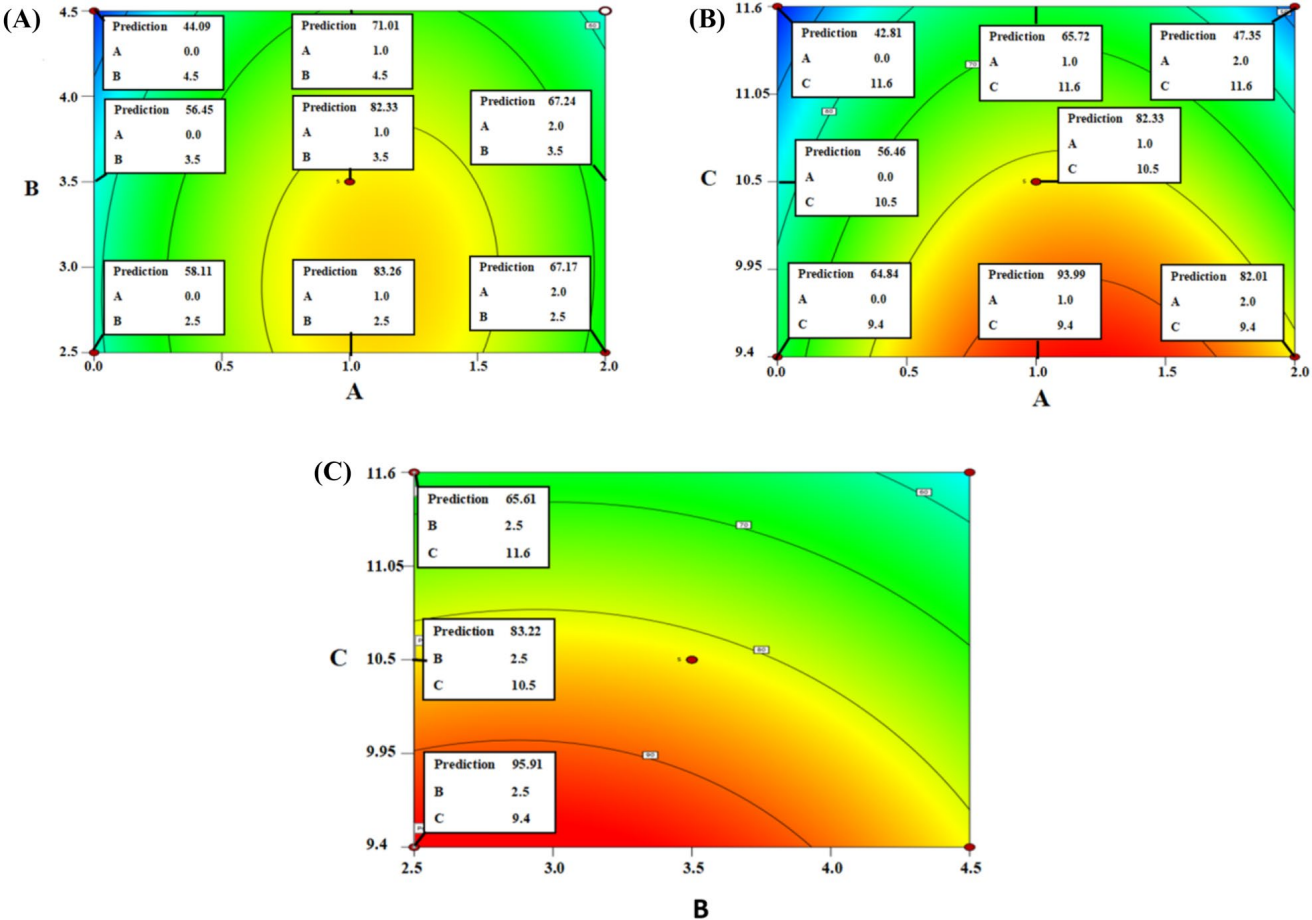
Contour plots which tracked the outcomes of manipulating the concentrations of EWP (**A**), and PEI (**B**), and the PEI pH (**C**). The response was the IE. Each plot considered the manipulation of only 2 factors whilst holding the remaining factor at its 0 level

**Fig. 6:**
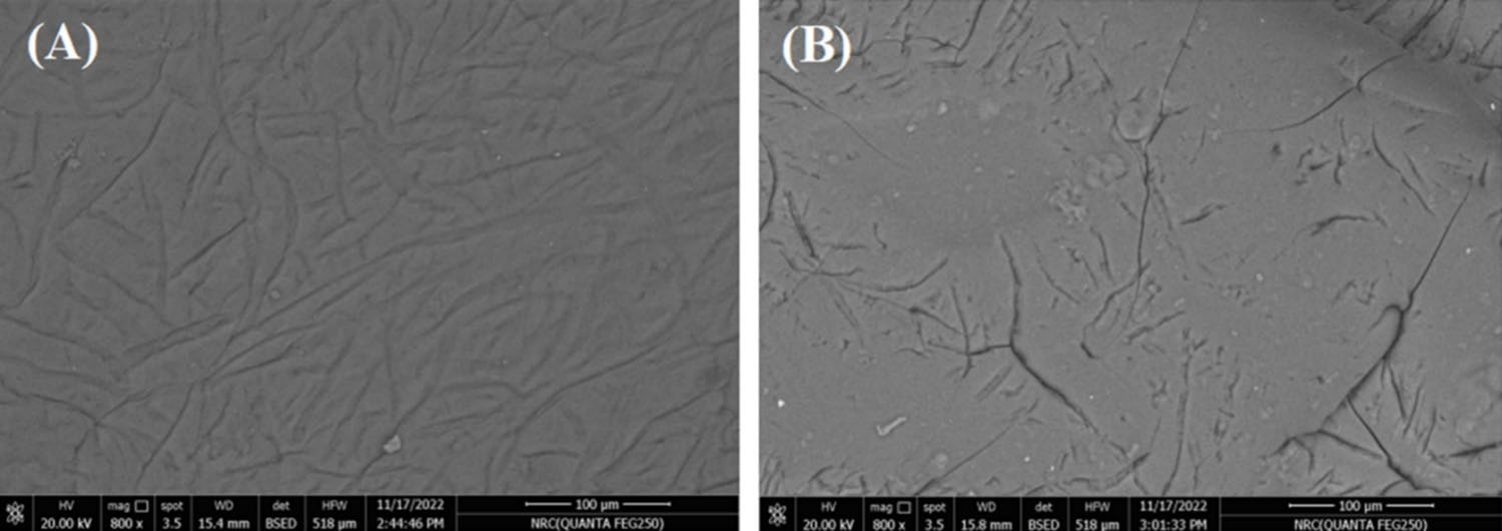
800X magnification of the **A** CP-EWP, and **B** CP-EWP/PEI/GA beads surfaces

The model *P*-value was 0.0144, and its R^2^ was 0.88, and this confirmed the model significance and adequacy to interpret the results, respectively. Supplementary Table (3) disclosed that the linear PEI pH term and the quadratic EWP concentration term were significant. The significance of blending EWP within the CP beads was also disclosed in Fig. 6A, which tracked the outcomes of manipulating the EWP (A) and PEI (B) concentrations whilst holding the PEI pH at its central (pH 10.5) value. At all tested PEI concentrations, blending 1% EWP would be beneficial and would raise the IE (Fig. 5A). For instance, 1.43, 1.46, and 1.61-fold raises would be recorded in the IEs if 1% EWP was blended into the 2.5, 3.5 and 4.5% PEI activated beads, respectively. Similar raises were observed in Fig. 5B, which tracked the outcomes of manipulating the EWP concentration (A) and PEI pH (C) at a fixed PEI concentration of 3.5% (central value). Blending 1% EWP into the CP beads, which were activated via pH 9.4, 10.5, and 11.6 PEI solutions, would raise the IE by 1.45, 1.46, and 1.53-folds, respectively (Fig. 5B). The optimal EWP concentration was shown to be 1%. Moreover, the optimal PEI processing conditions were 2.5% PEI concentration and 9.4 pH. Raising the PEI pH beyond the optimal 9.4 would diminish the IE. For instance, −1.15 and −1.46-fold IE diminutions would be induced if the pH of the 2.5% PEI solution, which was employed to activate the CP-1% EWP beads, was raised from 9.4 to 10.5 or 11.6, respectively (Fig. 5C). The optimal settings were the settings of run 6 which presented IY of 93.87% and possessed the largest IE of 94.95% (Table 7).

**Fig. 7:**
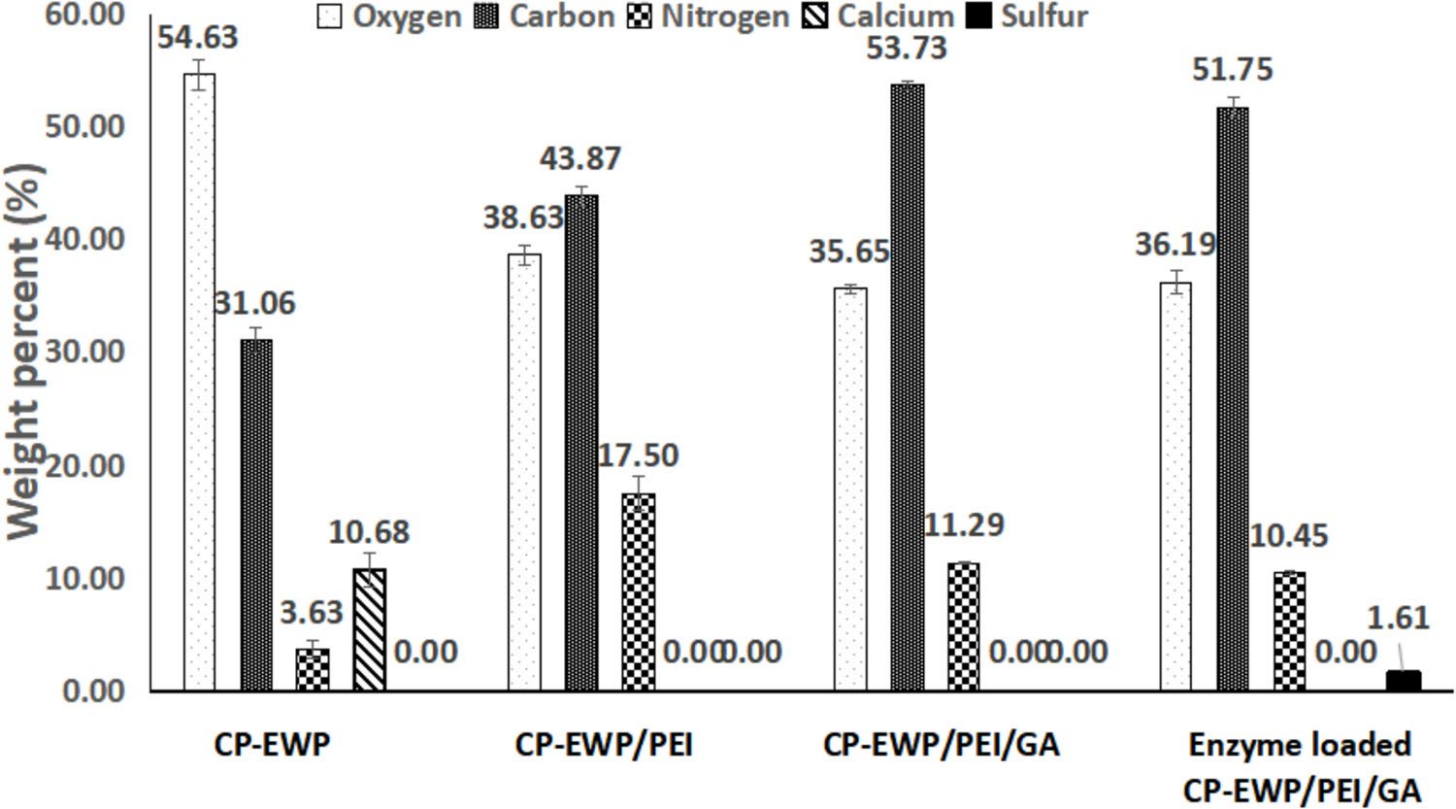
Elements present in the CP-EWP, CP-EWP/PEI, CP-EWP/PEI/GA, and the enzyme loaded CP-EWP/PEI/GA beads

SEM analysis revealed that the surface irregularities in CP-EWP beads were diminished after activation with PEI and GA, indicating successful modification (Fig. 6). Moreover, the elemental analysis of the CP-EWP beads disclosed that the beads offered 3.63% nitrogen. This nitrogen percent was raised to 17.5% after beads-interaction with PEI. The interaction with PEI also raised the beads carbon percent from 31.06 to 43.87%. As regards to the GA binding, it raised the CP-EWP/PEI/GA beads carbon content to 53.73%. Enzyme binding slightly reduced carbon and nitrogen percentages and introduced sulfur (1.61%) in the enzyme-loaded beads (Fig. 7).

**Fig. 8:**
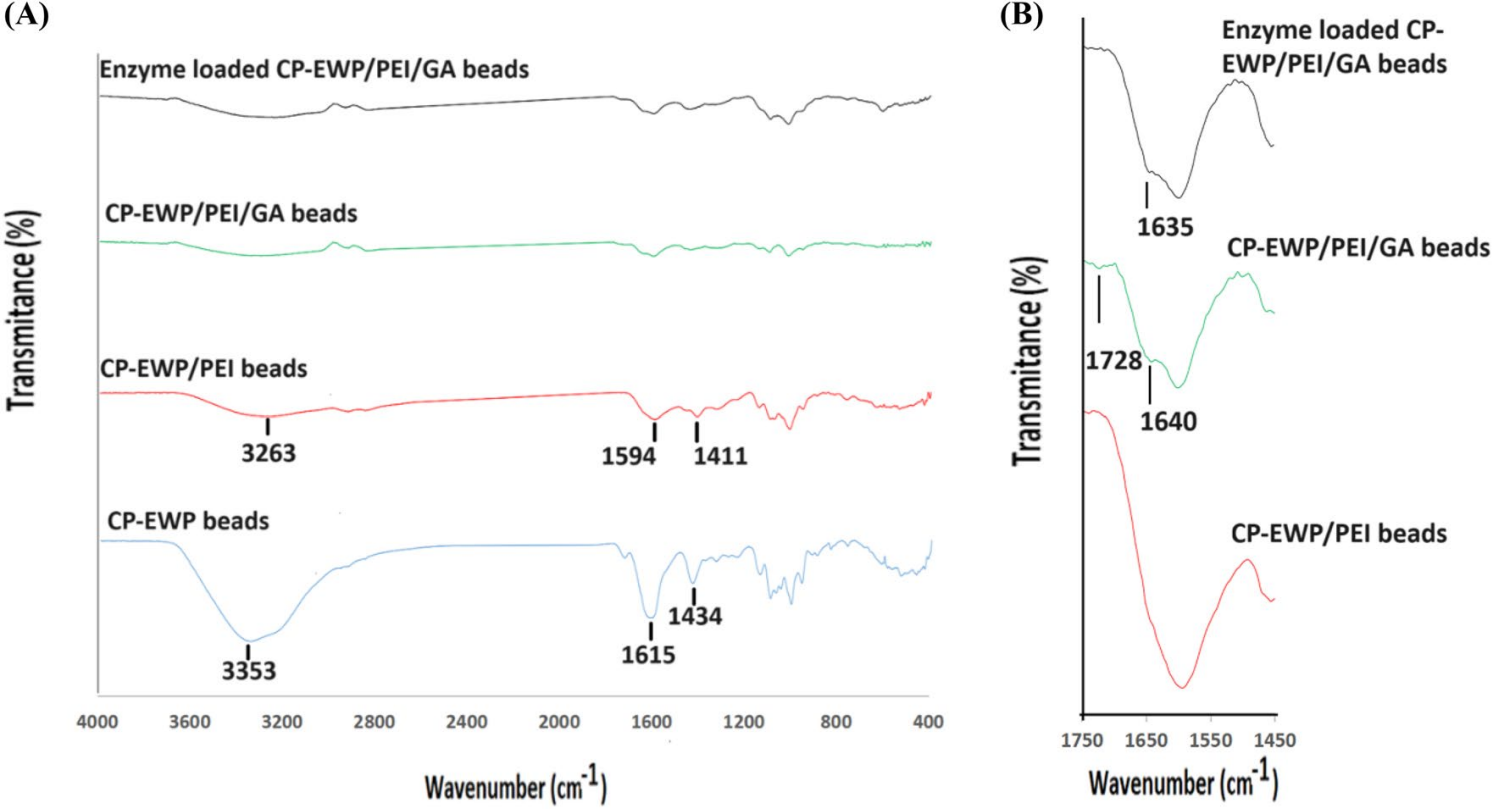
**A** FTIR spectra of the CP-EWP, CP-EWP/PEI, CP-EWP/PEI/GA, and the enzyme loaded CP-EWP/PEI/GA beads within the 4000–400 cm^−1^ range, **B** FTIR spectra of the CP-EWP/PEI, CP-EWP/PEI/GA, and the enzyme loaded CP-EWP/PEI/GA beads within the 1750–1450 cm^−1^ range

FTIR analysis of CP-EWP beads showed a broad OH stretching band at 3353 cm⁻^1^, with peaks at 1615 cm⁻^1^ and 1434 cm⁻^1^ corresponding to carboxylate and amino acid groups (Fig. 8A). After PEI activation, these bands shifted to 3263 cm⁻^1^, 1594 cm⁻^1^, and 1411 cm⁻^1^, reflecting ionic interactions between carboxylate groups and PEI amines. New bands at 1728 cm⁻^1^ and 1640 cm⁻^1^ in CP-EWP/PEI/GA beads were assigned to GA aldehyde groups and Schiff bases, respectively. The enzyme-loaded beads lacked the aldehyde band, confirming its consumption during enzyme binding (Fig. 8B).

**Fig. 9:**
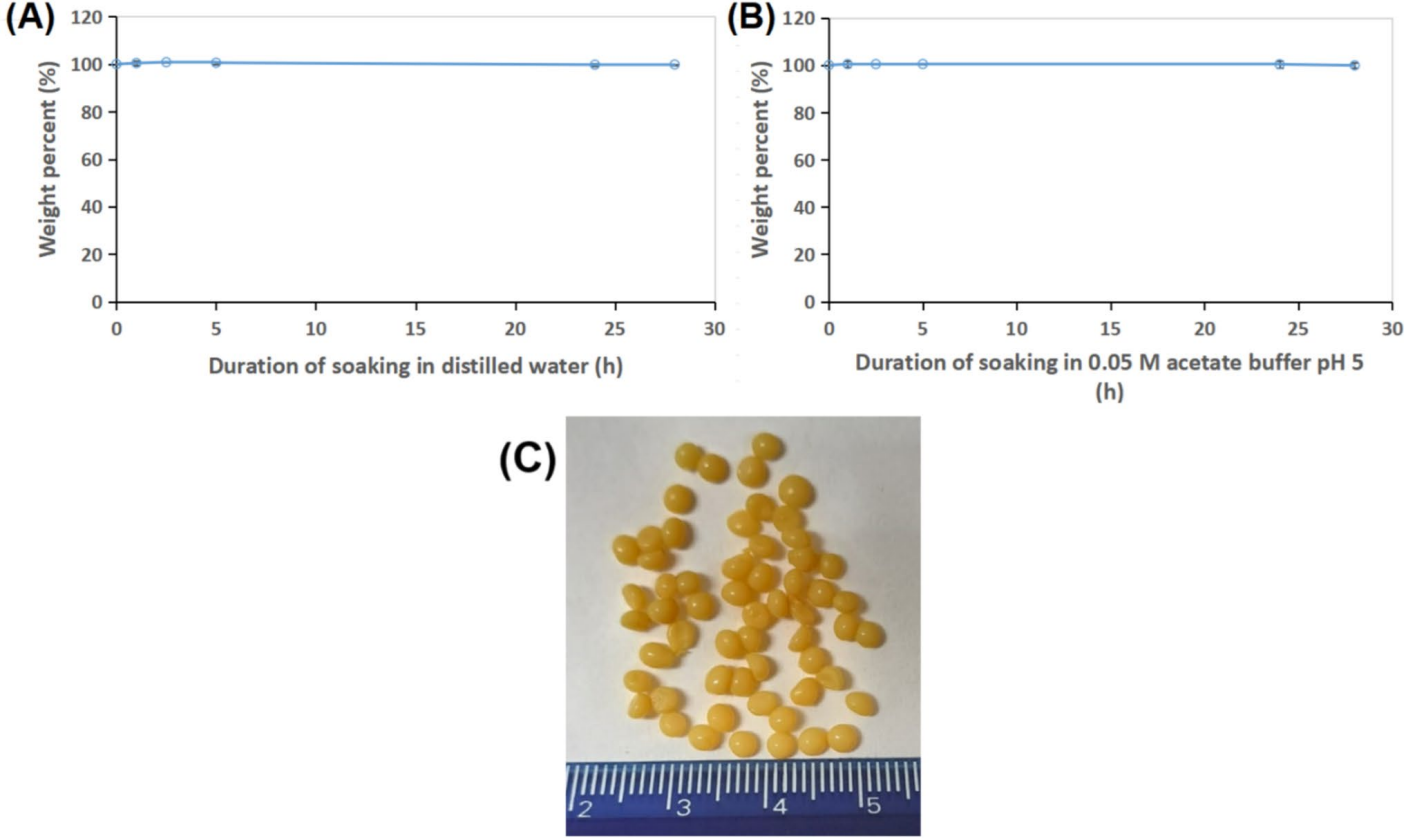
The weight percents kept by the CP-EWP/PEI/GA beads following their immersion in (**A**) distilled water and (**B**) 0.05 M acetate buffer pH 5

The CP-EWP/PEI/GA beads mechanical stability was also inspected, and it was shown that the CP-EWP/PEI/GA beads kept 98.27 ± 0.88% of their inceptive weight after being vortexed for 3 min and 20 s with minute glass beads. Moreover, the CP-EWP/PEI/GA beads stability in aqueous media was inspected. The beads were soaked in either distilled water or 0.05 M acetate buffer pH 5. In both cases, no swelling was observed even after 28 h of immersion in the respective aqueous medium. The beads weight fluctuated slightly amongst 99.84% and 100.81% (Fig. 9A, B). Figure 9C also revealed that the CP-EWP/PEI/GA beads were more or less homogeneous in size.

**Fig. 10:**
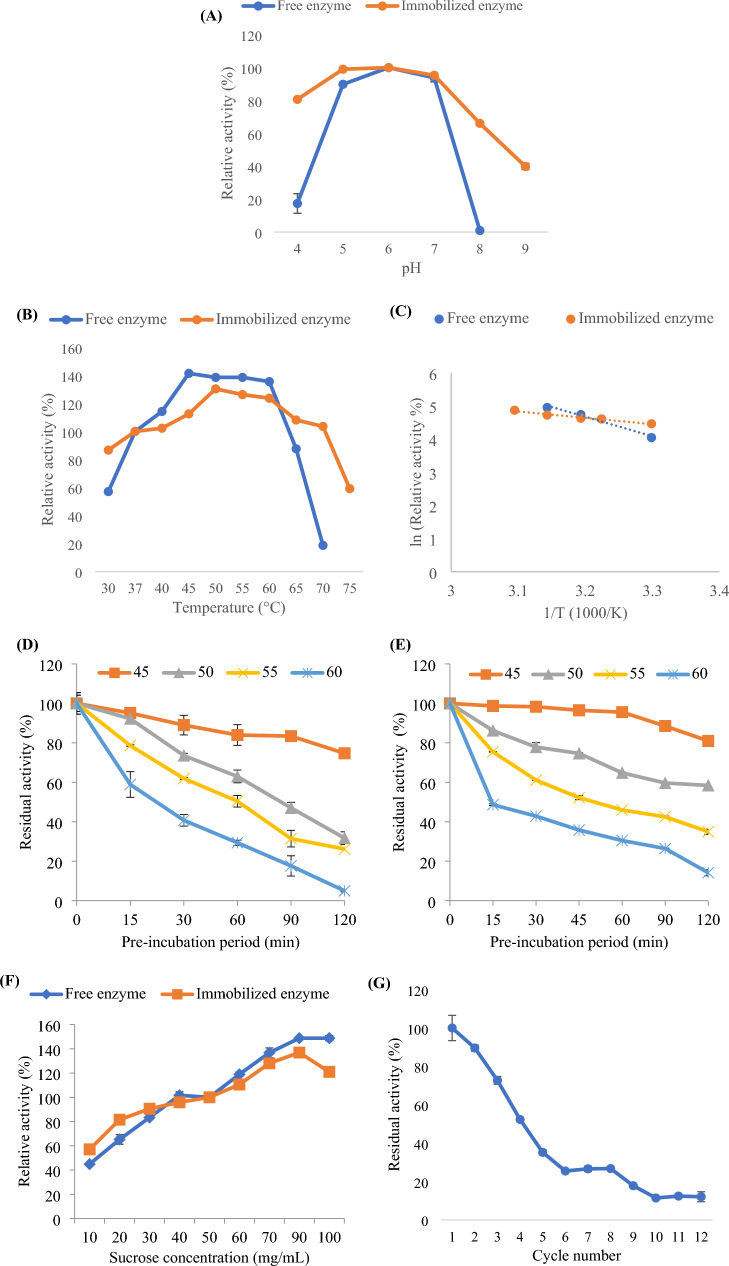
Characterization of the immobilized enzyme; the effect of **A** pH, **B** temperature on the enzyme activity, **C** thermal activation Arrhenius plot, thermal stability study of **D** the free and **E** the immobilized enzyme, **F** the effect of sucrose concentration and **G** reusability study

The enzyme activity was characterized in respect to pH, temperature, and substrate concentration influences. The enzyme optimum activity was found to be between pH 5 and neutral pH. Additionally, the immobilized enzyme was active in alkaline environments; at pH 8 and pH 9, it was 66% and 40% active, respectively (Fig. 10A). In respect to the temperature influence, the free and immobilized enzymes had optimum activities between 45 and 60 ºC and 50–60 ºC, respectively. Moreover, the immobilized enzyme possessed activity at higher temperatures, it retained its complete initial activity at 70 ºC and retained 59% at 75 ºC (Fig. 10B). Furthermore, the activation energy, which is needed by the immobilized enzyme to conform its active site and create the enzyme–substrate-complex, was reckoned from the Arrhenius plot (Fig. 10C) as 15.914 kJ mol^−1^. This was approximately the third of that estimated for the free form (48.881 kJ mol^−1^). The immobilized enzyme thermal stability study was examined by determining its remaining activity following its pre-incubation at altered temperatures (45 to 60 ºC) for variable periods. From the results (Fig. 10D, E), it was revealed that the immobilized enzyme K_d_ values were lesser than those of the free form and consequently, higher T_1/2_, and *D*-values were estimated for the immobilized form (Table 8). By examining the impact of sucrose concentration on the enzyme activity, a gradual rise in the activity of the enzyme was observed by elevating the concentration up to 90 mg mL^−1^ for the free and immobilized samples without detecting any significant increase at higher concentration (Fig. 10F). The enzyme reusability testing showed that during the fourth cycle, it maintained over 50% of its activity (Fig. 10G). Moreover, after a month of chilling storage, the immobilized enzyme was still fully active when the beads were submerged in distilled water.

**Table 8:** Thermo-kinetic parameters

Temperature (^ο^C)	Kd (min^−1^)		T_1/2_ (min)		*D*-value (min)	
	Free	Immobilized	Free	Immobilized	Free	Immobilized
45	0.0022	0.0017	315.07	407.73	1046.63	1354.46
50	0.0093	0.0045	74.53	154.03	247.59	511.69
55	0.0112	0.0081	61.89	85.57	205.589	284.27
60	0.0222	0.0135	31.22	51.34	103.72	170.56

## Discussion

Honey is composed primarily of carbohydrates, including monosaccharides such as fructose (32–44%) and glucose (23–38%), along with various complex sugars (5–15%) (Meo et al. 2017). This sugar-rich composition makes honey an ecological niche for diverse microorganisms, which can originate from bees and their surrounding environment, or contamination during processing and storage (Luca et al. 2024). Some of these microorganisms have been identified as probiotics, offering beneficial health properties, while others have demonstrated the ability to utilize sucrose for exopolysaccharide (EPS) production (María et al. 2021; Wahab et al. 2024).

In the current study, a honey isolate furtherly identified as *Bacillus subtilis* was examined for the production of exo-polysaccharide under submerged fermentation of sucrose. Ethanol precipitation of the cell-free culture indicated the formation of a complex carbohydrate with molecular weight of 75 KDa in which glucose was its monosaccharide constituent. *Bacillus subtilis* has been reported as an efficient source for the production of exo-polysaccharides with promising biological activities (Abdel-Wahab et al. 2022, Sathishkumar et al. 2021, Swartzendruber et al. 2019 and Yan et al. 2024). In the current study, four selected polysaccharides of different molecular weights (51, 75, 100 and 168 KDa), produced by the fermentation of sucrose under various cultural and nutritional conditions using the isolated *Bacillus subtilis,* was estimated as dextrans based on FTIR, ^1^H and ^13^C NMR analysis. Dextrans are α-glucan polysaccharides mainly composed of glucose chains of 1,6 linkages with some α−1,2, α−1,3 and α−1,4 branches (Jeanes et al. 1954). The investigated dextrans showed antifungal action against a strain of *Candida albicans* that was resistant to fluconazole, antibacterial activity against *Staphylococcus aureus*, and a variable prebiotic response with *L. casei*. Moreover, the examined dextrans of higher molecular weight (100 and 168 KDa) possessed fibrinolytic and anticoagulant activity equivalent or higher to that of commercial standards. Previously, dextrans and their derivatives had been reported to possess various biological activity including prebiotic, antimicrobial, fibrinolytic, antioxidant and immunomodulatory, in which a certain molecular weight possessed the optimal activity (Amaretti et al. 2020, Du et al. 2023, Esawy et al. 2016, Pramudito et al. 2024, Soeiro et al. 2016, Vrzoňová et al. 2023, Yilmaz et al. 2022).

Dextransucrases are glucansucrases that catalyze the synthesis of dextran (Monchois et al. 1999). In the current study, the produced glucansucrase in the cell-free culture was estimated as 3.257 U/mL that was furtherly increased to 38.4 U/mL by the use of sucrose of 20% as the carbon source and urea of 0.06% as the nitrogen source with the addition of wheat flour of 8% that incubated at 30 ºC for 18 h. Moreover, statistical designs including PB and BB designs were applied to optimize the fermentation process. In PB, the impact of seven variables was examined in which the significant variables were identified. Preliminary screening using PB enables the quick identification of the influencing variables with saving time and effort in addition to reducing the experimental cost. Furtherly, the significant variables were optimized by applying BB design. This aided in figuring out the ideal concentrations of each component and comprehending the interactions between variables (Ismail et al. 2024a,b; Hassan and Ismail 2021; Helmy et al. 2024). Following statistical optimization, the glucansucrase activity was increased to 49.485 U/mL. This result was more than tenfold higher than *Leuconostoc mesenteroides* (L.M.CICC-20724) produced activity (4.2 U/mL) (Hou et al. 2018) and 4.7 U/mL reported for *Weissella cibaria* RBA12 (Baruah and Goyal 2015). In addition, it was higher than the optimized 10 U/mL reported for *Pediococcus pentosaceus* CRAG3 (Shukla and Goyal 2012) and 6.26 U/mL reported for *Leuconostoc mesenteroides* DRP 105 (Du et al. 2017).

In general, enzyme immobilization has shown promising efficiency in protecting enzymes from degradation or inactivation in harsh operating conditions as well as improving its operational stability and consequently, extending their industrial exploitation (Wahba et al. 2024). Concerning glucansucrases, the applied immobilization protocols led to low IY and IE (da Silva et al. 2019, 2020; Graebin et al. 2016, 2018). In the current study, immobilization of the 70% ammonium sulphate precipitated glucansucrase on CP-EWP/PEI/GA beads was examined. Initially, the CP-EWP/PEI/GA beads were optimized via the applying of BB design in order to efficiently immobilize the produced enzyme. In the BB design, the EWP concentration that should be blended within the CP beads as well as the PEI optimal concentration and PEI pH were examined. Noteworthy, the inspected PEI pH and concentration ranges were selected to comprise the optimal settings for the formerly fabricated pectin immobilizers, such as the CP/PEI/GA (pH 10.55, 3.49%) (Wahba 2016); CP-carrageenan/PEI/GA (pH 9.58, 3.11%) (Wahba 2021), the amidated pectin/PEI/GA (pH 9.65, 3.4%) (Saleh et al. 2023). It was disclosed that the optimal EWP concentration, which should be blended within the CP beads, was 1%. This 1% EWP (4.5–4.7 pI (Lu et al. 2020; Razi et al. 2023)) raised the anionic attributes of the beads and boosted their ionic-interactions with the cationic PEI. Hence, the performance of the activated CP-EWP beads improved and the IE was raised. Noteworthy, the 1% EWP concentration was close to the 1.01% SPI concentration, which was formerly selected to be blended within the gellan gum beads during their PEI/GA activation (Wahba et al. 2022a). Noteworthy, raising the EWP concentration to 2% would reduce the IE at all tested PEI concentrations and pHs. This could be regarded to the excessive rise in the beads anionic attributes, which might have induced repulsions within the beads construction and impaired their performance and their reactivity with PEI.

The optimal PEI processing conditions were 2.5% PEI concentration and 9.4 pH. These values were proximate to the 3.11% concentration/9.58 pH, the 3.4% concentration/9.65 pH, and the 2.95% concentration/8.67 pH settings which were recorded as optimal during the PEI processing of the CP-carrageenan, the amidated pectin, and the CP-agar beads respectively (Saleh et al. 2023; Wahba 2020b, 2021). Noteworthy, raising the PEI pH beyond the optimal 9.4 would diminish the IE. Raising the PEI pH dwindles its presented cationic entities (Borkovec and Koper 1997). Thus, its ionic interactions with the anionic entities of the CP-EWP beads would be less efficient upon raising the pH, and this would eventually dwindle the efficiency of the activated CP-EWP immobilizers and dwindle the IE. The optimal conditions (EWP concentration of 1% and PEI processing conditions of 2.5% PEI concentration and 9.4 pH) presented immobilization yield of 93.87% and possessed IE of 94.95%.

Furthermore, characterization of the constructed beads was carried out on the base of SEM, elemental and FTIR scrutinization. The SEM disclosed that the surface irregularities of the CP-EWP beads were diminished after their PEI/GA activation. Likewise, the surface irregularities of the SPI-alginate-gellan gum beads and the gum tragacanth-agar disks were diminished after their PEI/GA activation (Wahba 2022b). The elemental scrutinization indicated that the CP-EWP beads offered 3.63% nitrogen content. This nitrogen confirmed the blending of the protein; EWP within the CP beads as pectin doesn^’^t comprise nitrogen (Einhorn-Stoll et al. 2021). After the inclusion of PEI, the beads nitrogen percent was raised to 17.5% and their carbon percent was also raised from 31.06 to 43.87%. These nitrogen and carbon raises confirmed the binding of PEI (H(NHCH_2_CH_2_)_n_NH_2_) to the CP-EWP beads. Likewise, the PEI binding to the SPI-alginate-gellan gum beads raised their nitrogen and carbon contents (Wahba et al. 2022a). Noteworthy, the GA binding raised the CP-EWP/PEI/GA beads carbon content to 53.73%. GA (C_5_H_8_O_2_) binding is usually coupled with the rise in the beads carbon content (Wahba 2020a, 2022b).

With respect to the FTIR inspection, the broad intense band, which was evident at 3353 cm^−1^ in the CP-EWP beads spectrum, represented the stretching-vibration of the plenteous OH residues (Hong et al. 2021) of CP. The bands at 1615 and 1434 cm^−1^ in the CP-EWP beads spectrum could be ascribed to the carboxylate forms (Nandiyanto et al. 2019) of the CP galacturonic acid entities and the carboxylic functionalities of the EWP amino-acids. Nonetheless, these bands were a bit higher than their reference ranges (1610–1550 and 1420–1300 cm^−1^, respectively (Nandiyanto et al. 2019). The slight up-shifting of these carboxylate bands could have occurred due to their overlapping with the primary and secondary amine bending bands (1650–1590; 1650–1550 cm^−1^, respectively (Nandiyanto et al. 2019)) of the EWP.

A clear diminution was observed in the area of the OH stretching-vibration band following the CP-EWP interaction with PEI. This band was also displaced to 3263 cm^−1^. Noteworthy, the area of the the gum tragacanth-agar disks OH stretching-vibration band was also varied following interaction with PEI (Wahba 2022b). This variation indicated the modification in the H-bonding of the specimen (Hong et al. 2021). Hence, it proved that H-bonds were created between the polysaccahride matrix and PEI (Wahba 2022b). The ionic interactions amidst the carboxylate entitities of the CP-EWP and the cationic PEI were also evident in the FTIR of the CP-EWP/PEI spectrum as the carboxylate bands were displaced from 1615 and 1434 cm^−1^ to 1594 and 1411 cm^−1^, respectively. This shifting could have also been caused by the overlapping between these carboxylate bands and the primary and secondary amine bending bands (1650–1590; 1650–1550 cm^−1^, respectively (Nandiyanto et al. 2019)) of PEI. Noteworthy, the stretching bands of the primary and secondary amine (3400–3380; 3360–3310 cm^−1^, respectively (Nandiyanto et al. 2019)) of PEI could have also been overlapped with the OH stretching vibration band. Similar overlapping was also observed in the spectrum of the gum tragacanth-agar/PEI specimen (Wahba 2022b).

Two new bands were evident in the CP-EWP/PEI/GA beads spectrum. A band at 1728 cm^−1^and a band at 1640 cm^−1^. The former band represented the GA aldehyde entities (Aldehyde; 1740–1725 cm^−1^ (Nandiyanto et al. 2019)) whereas the latter band represented the Schiff base established after the interaction of GA with the amino entities of PEI (-C = N-; 1690–1590 cm^−1^ (Nandiyanto et al. 2019). Noteworthy, the band representing the GA aldehyde entities was formerly reported to appear at around 1727–1721 cm^−1^ (Wahba 2022b). Nonetheless, the aldehyde band wasn^’^t detected in the enzyme loaded beads. The aldehyde entities were consumed in the interaction with the enzyme. As regards to the Shciff base band, it was still evident in the enzyme loaded beads at 1635 cm^−1^ as more Schiff base was formed following the GA-enzyme interactions.

The CP-EWP/PEI/GA beads mechanical stability was verified as the beads kept 98.27 ± 0.88% of their inceptive weight after being vortexed for 3 min and 20 s with minute glass beads. This mechanical stability surpassed that of the CP-agar/PEI/GA immobilizers whose beads kept only 81.97% of their inceptive weight after being analogously vortexed (Wahba 2020b). The CP-EWP/PEI/GA beads were also stable in aqueous media and didn^’^t swell despite of being soaked for 28 h. The heightened physical stability of the CP-EWP/PEI/GA beads could be regarded to the PEI/GA processing. The PEI formulated a somewhat hard shell amidst the beads, and this shell was further reinforced by GA cross-linking (Wahba 2016).

By evaluating the impact of pH and temperature on the activity of the immobilized enzyme in compare to the free form, a significant improvement in the enzymatic activity at higher pHs and temperatures was estimated by immobilization. It has been documented that immobilization serves as a defense against the molecular conformational changes of enzymes brought by harsh conditions including alkaline pH and high temperature (da Silva et al. 2019). Moreover, the immobilized enzyme in the current study possessed a significant decrease in activation energy, approximately the third of that of the free form, with improved thermal and operational stability. These criteria are preferred for the industrial application of the immobilized enzymes as they can reduce the total operating costs. In addition, high operating temperatures reduce the growth of microbial contaminates and consequently protect the final product (da Silva et al. 2019, 2020).

## Conclusion

In the current research, a honey isolate identified as *Bacillus subtilis* was estimated as a glucansucrase producer via the fermentation of sucrose in which the synthesized glucan was dextran in nature. The synthesized glucan possessed prebiotic, antimicrobial, fibrinolytic and anticoagulant activities. Moreover, the produced glucansucrase was statistically optimized by applying PB and BB designs. Furthermore, CP-EWP/PEI/GA beads were used to immobilize it; BB design was used to estimate the ideal parameters for bead construction, and the maximum estimated immobilization yield and efficiency were 93.87 and 94.95%, respectively. The immobilized enzyme had a low activation energy and increased activity at high temperatures and alkaline pHs. In addition, the immobilized enzyme retained over 50% of its activity at the fourth cycle with the retaining of its activity for more than one month under cooling conditions when immersed in distilled water, indicating improved operational stability.

## Supplementary Information

Below is the link to the electronic supplementary material.

Supplementary file1 (DOCX 1842 KB)

## Data Availability

The data that support the findings of this study are openly available.
