# Supplementary material for: Bacillus subtilis EGY1 glucansucrase: optimization, characterization and immobilization using activated carrier of pectin-egg white protein beads
Source: 3 Biotech. 2025 Dec 8;16(1):16. doi: 10.1007/s13205-025-04609-7 (PMC12686235; doi:10.1007/s13205-025-04609-7)
Supplement: Supplementary file 1 — Supplementary file1 (DOCX 1842 KB) [file 13205_2025_4609_MOESM1_ESM.docx]

| **D1**  **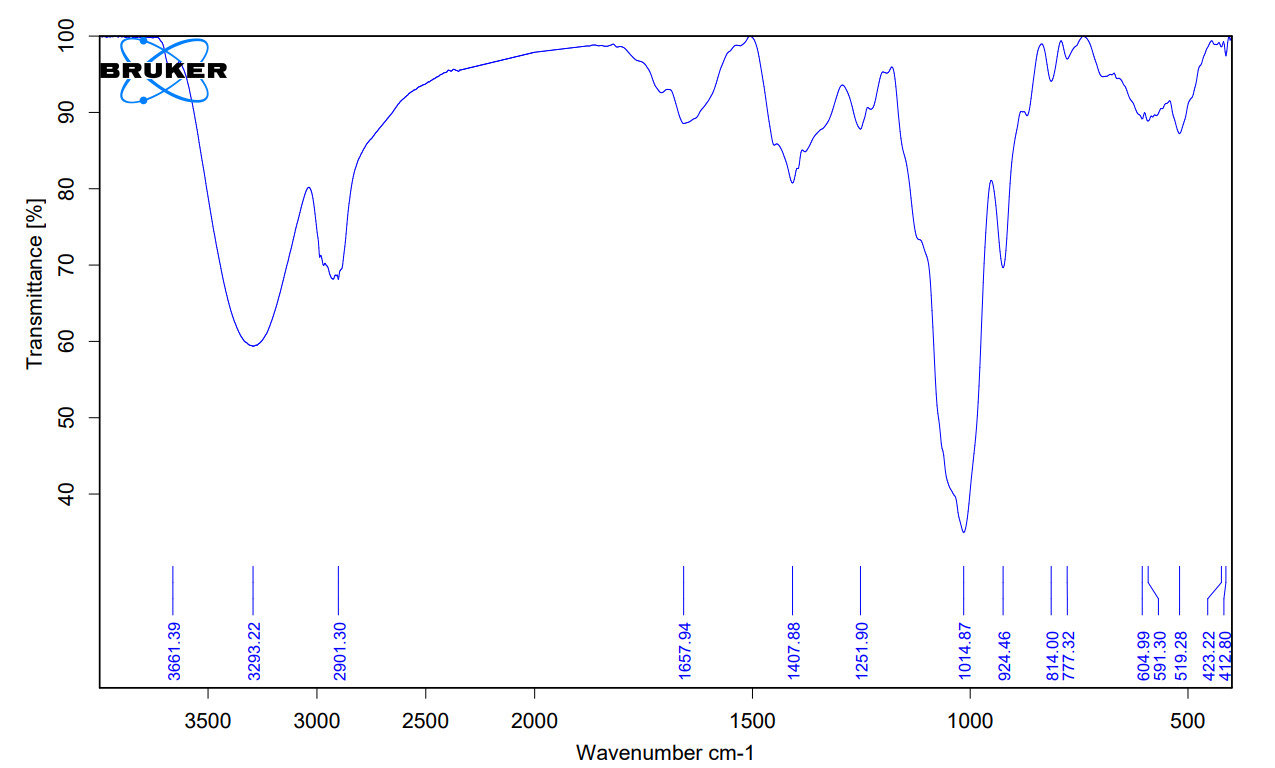** |
| --- |
| **D2**  **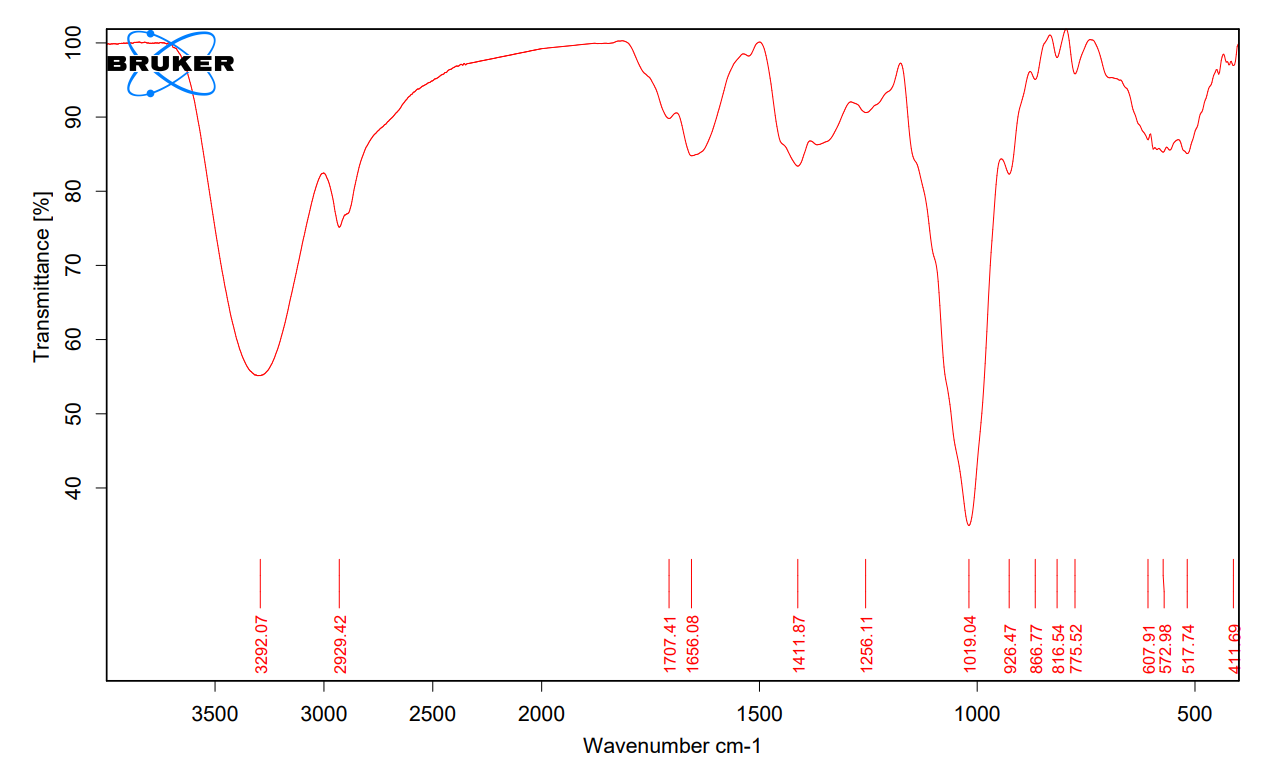** |
| **D3**  **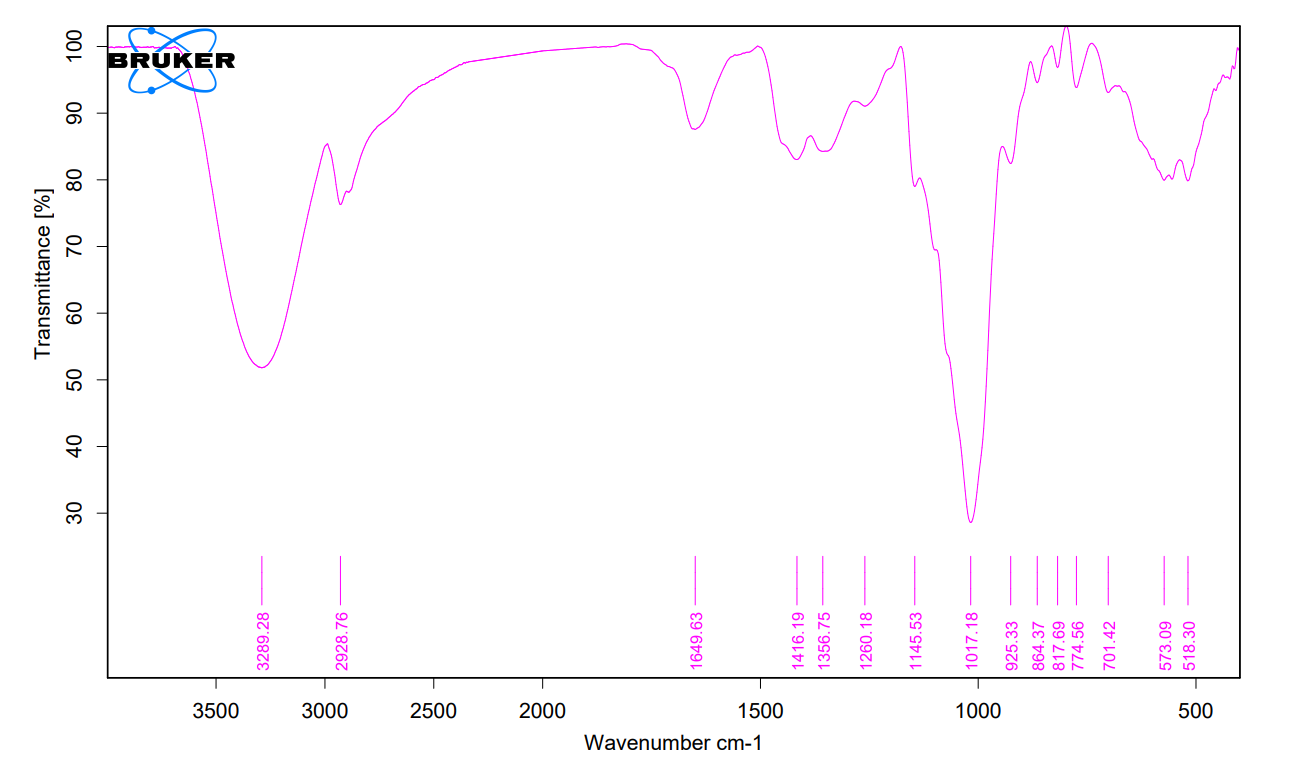** |
| **D4**  **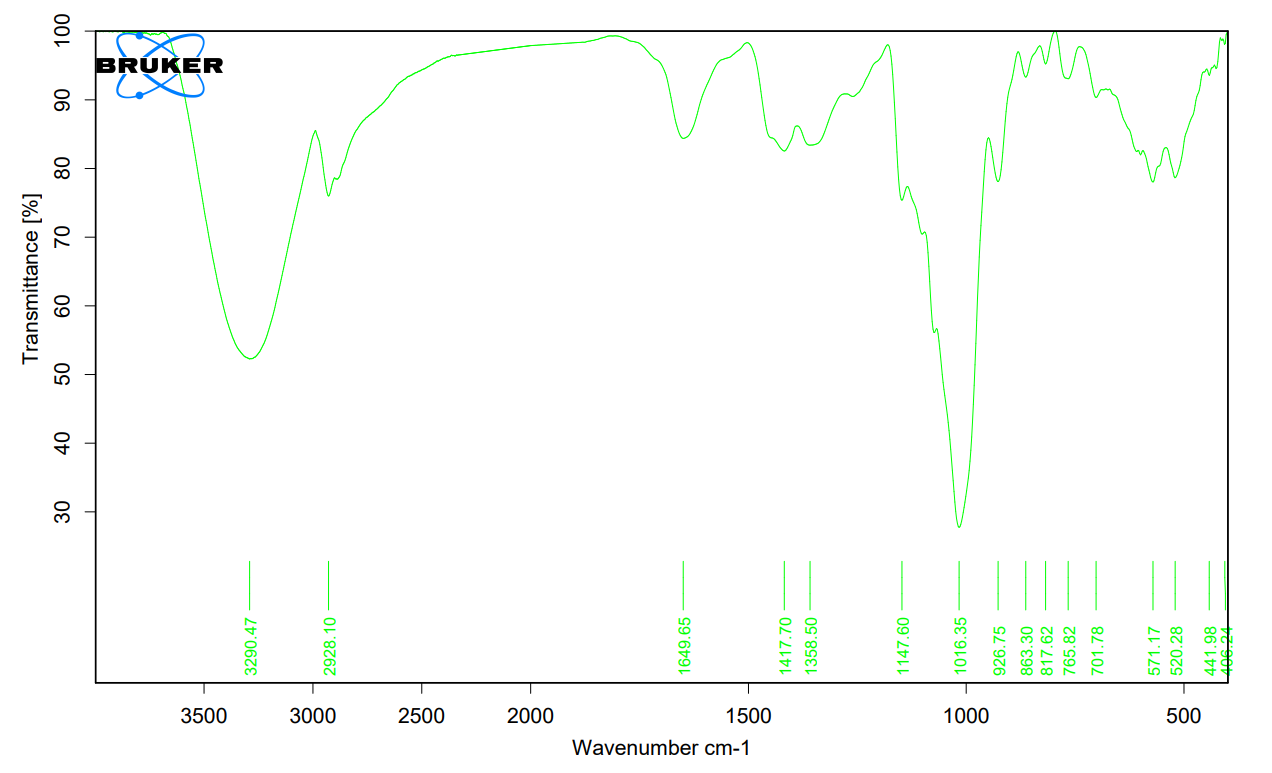** |

Supplementary figure (1): FTIR analysis of the precipitated glucans in which D1, D2, D3 and D4 were glucans of molecular weight 51, 75, 100 and 168 KDa, respectively.

| **D1**  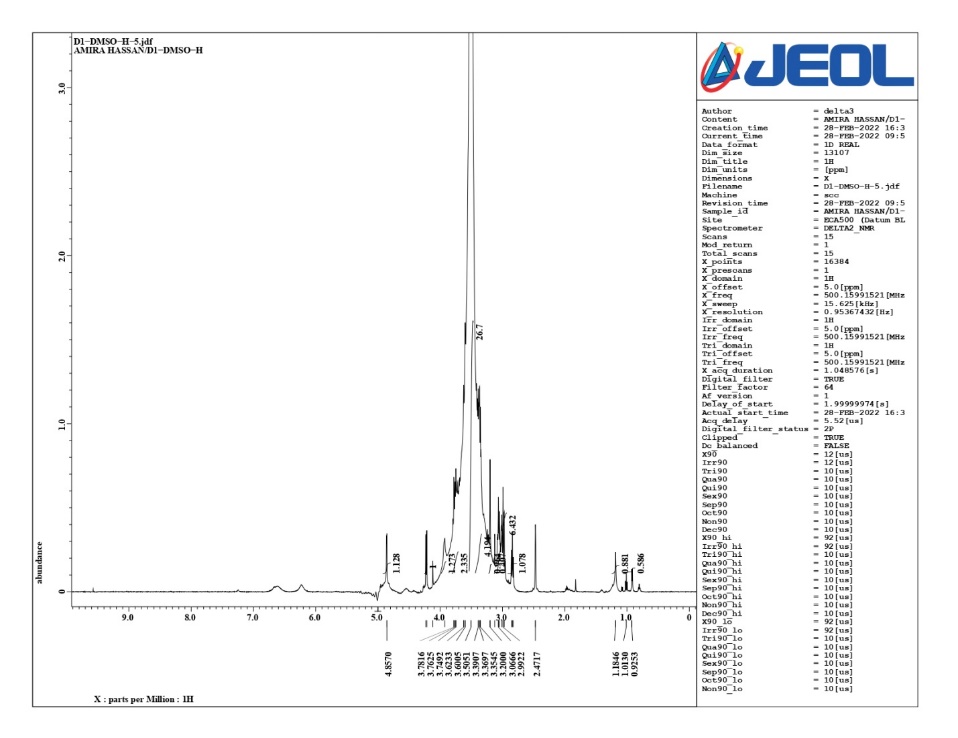 | 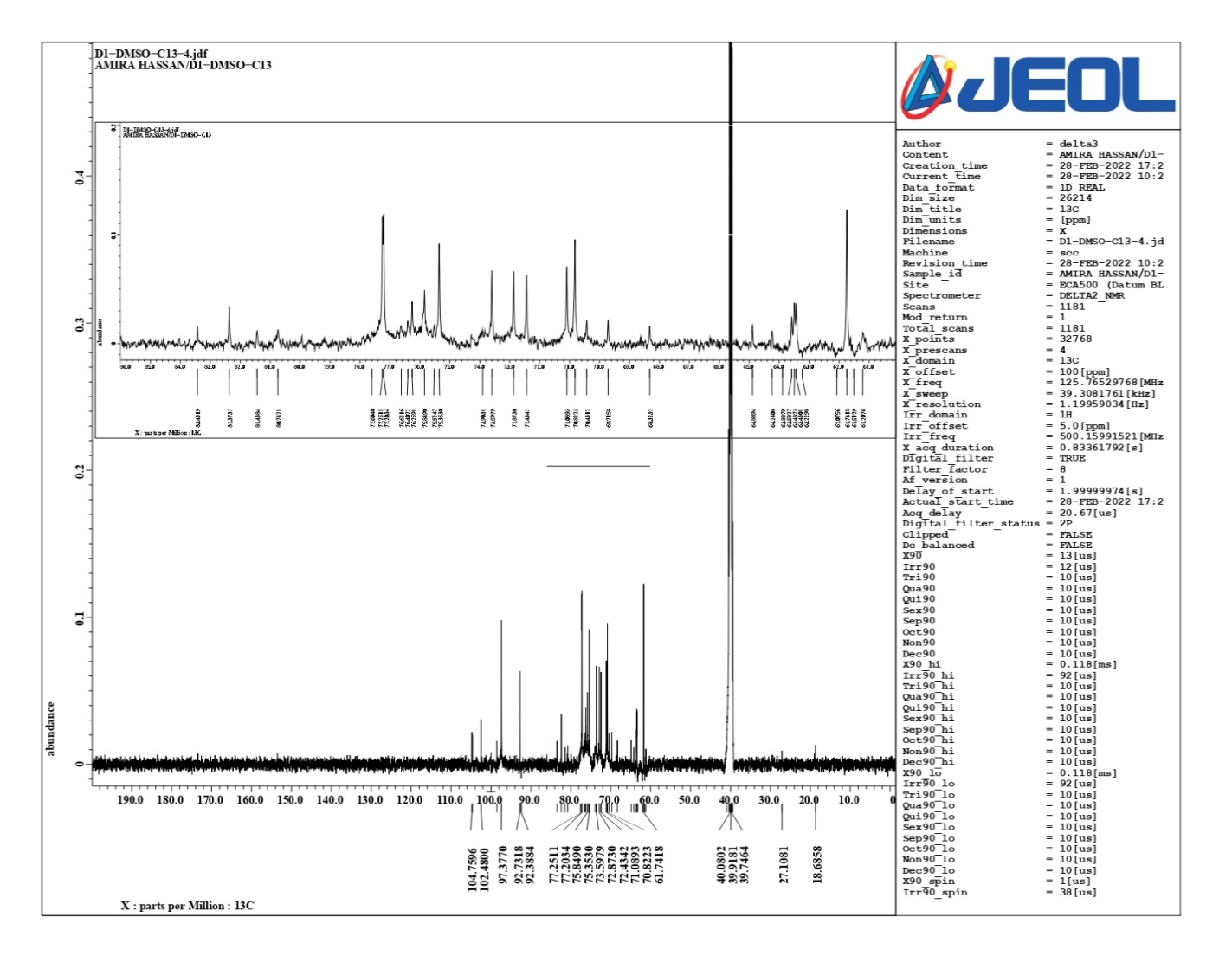 |
| --- | --- |
| **D2**  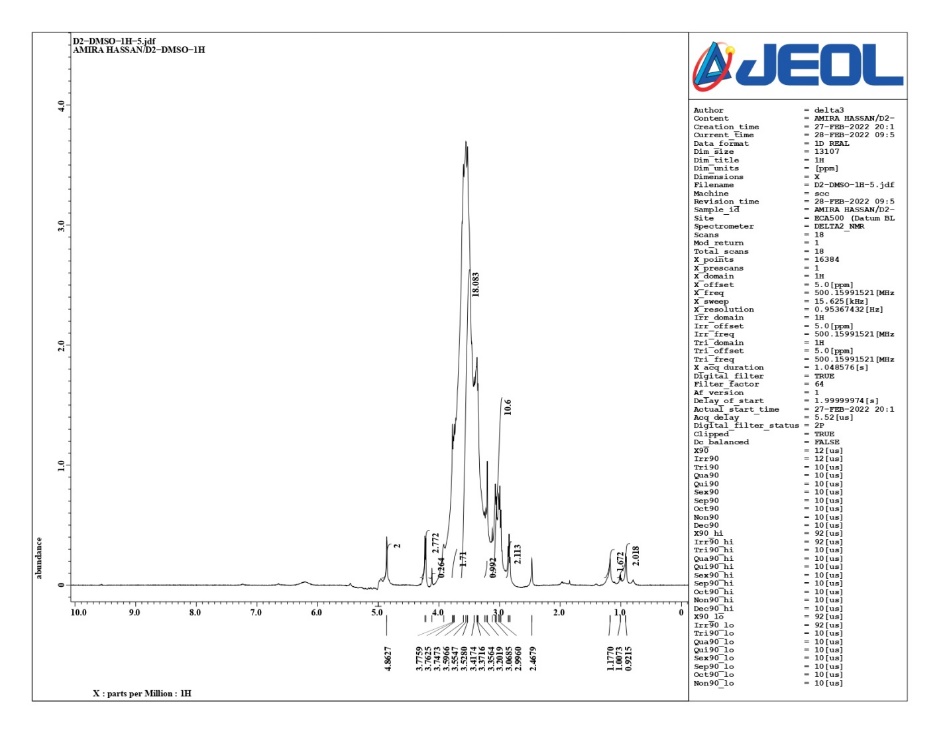 | 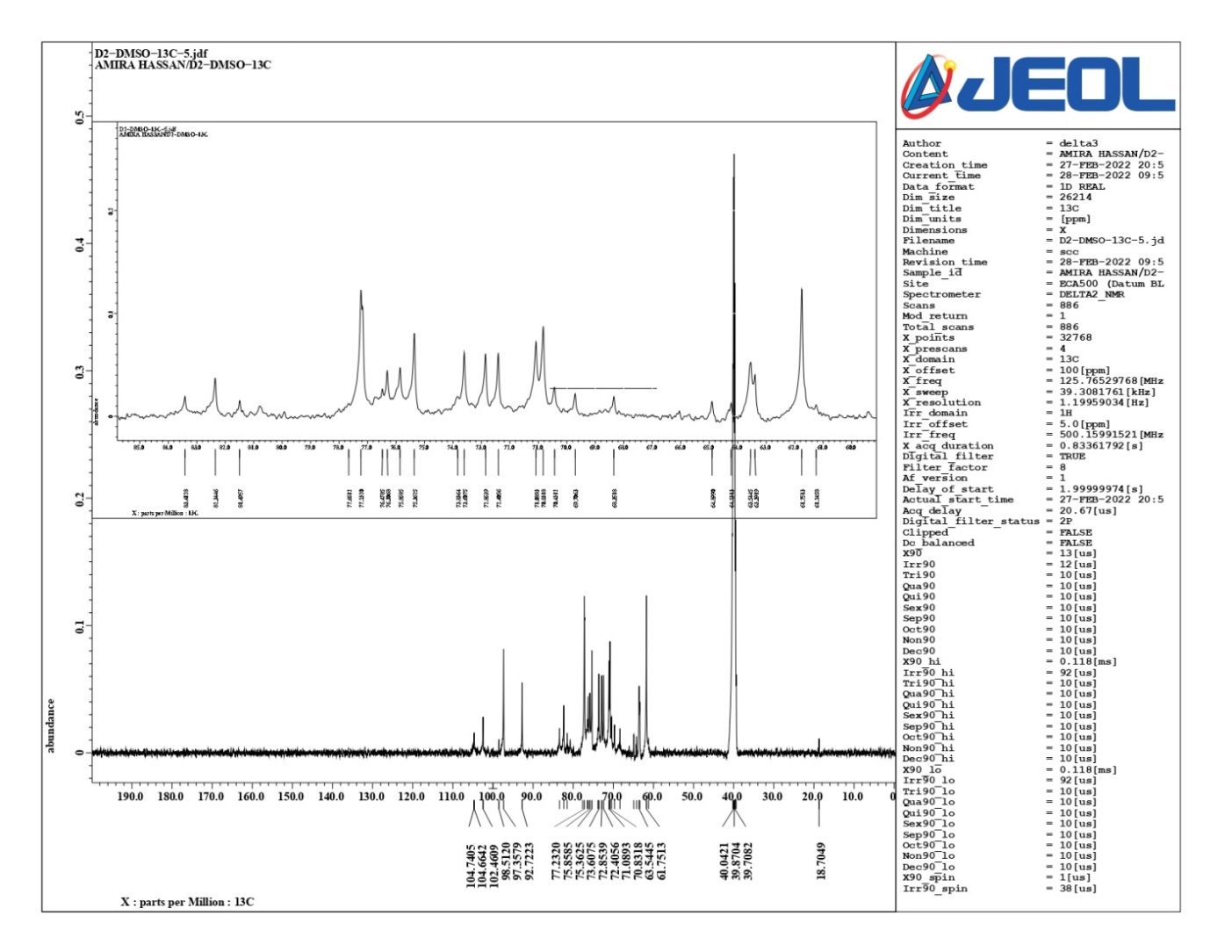 |
| **D3**  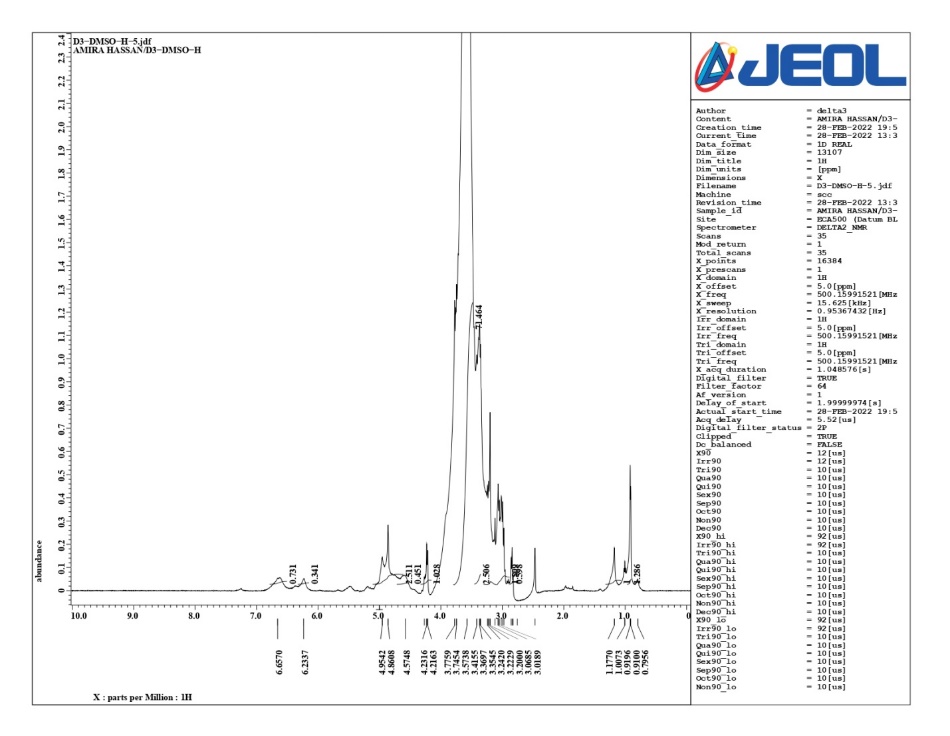 | 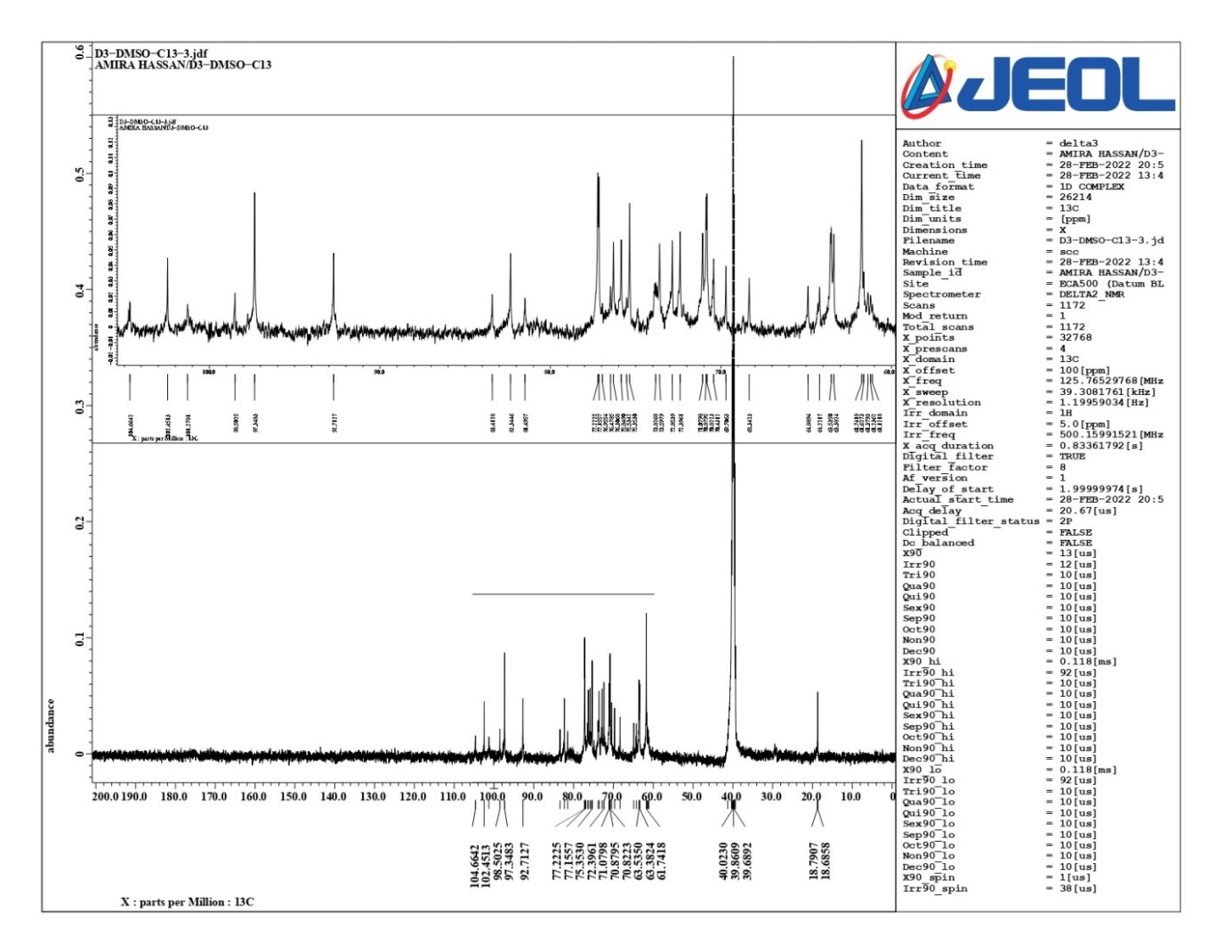 |
| **D4**  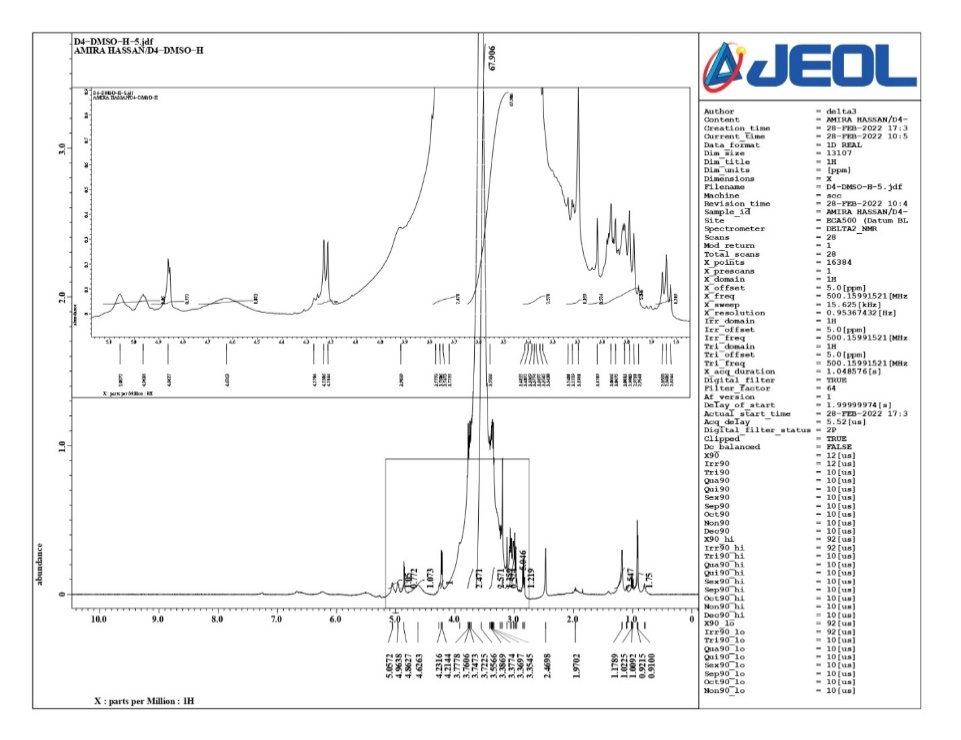 | 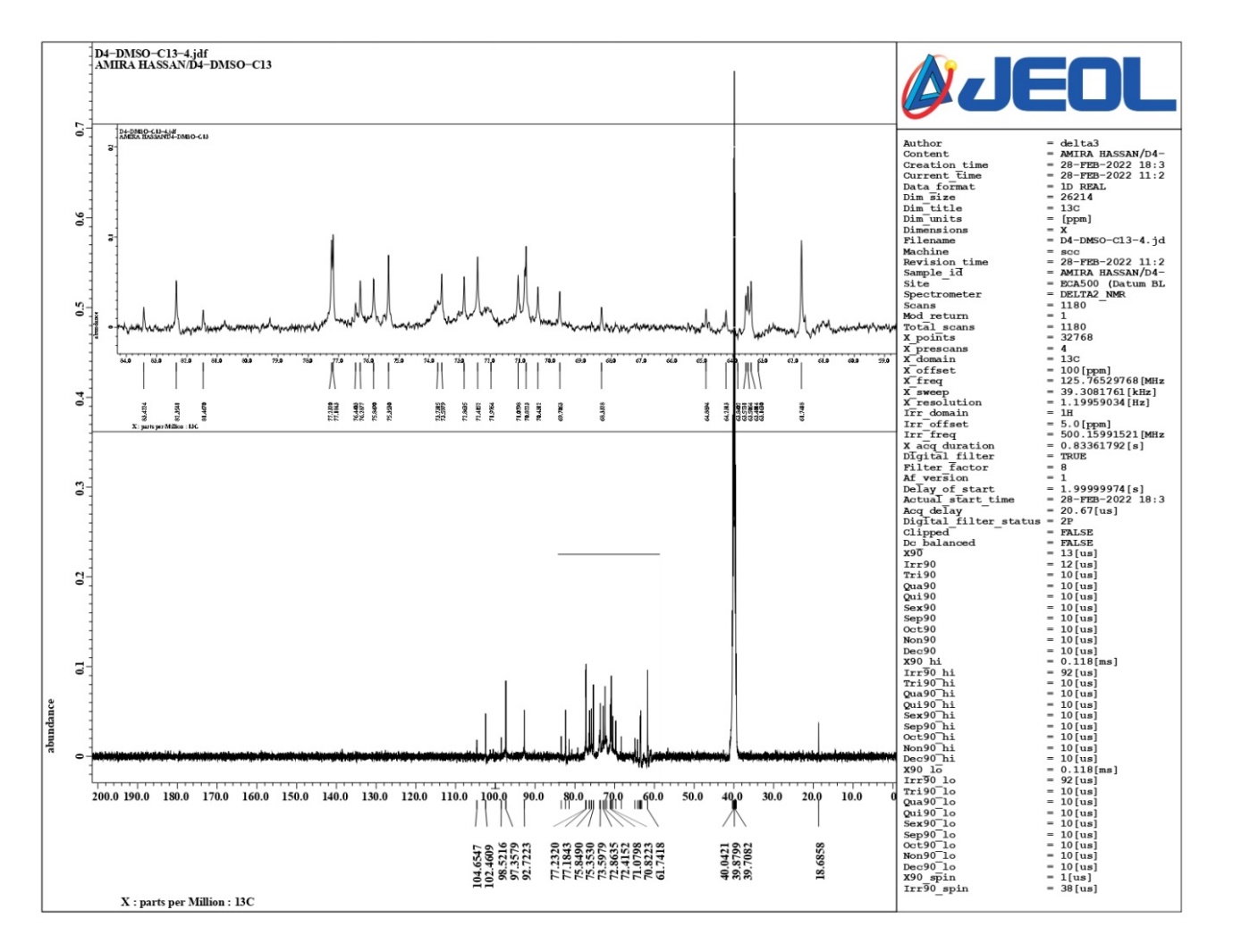 |

Supplementary figure (2): ^1^H and ^13^C NMR of the precipitated glucans in which D1, D2, D3 and D4 were glucans of molecular weight 51, 75, 100 and 168 KDa, respectively.

**Supplementary table (1): Regression analysis of Plackett-Burman design**

| Variables | Enzyme activity analysis | | | |
| --- | --- | --- | --- | --- |
|  | **Coefficient** | **Standard error** | ***t*-statistics** | ***P*-value** |
| Intercept | 43.6789 |  |  |  |
| Sucrose concentration (%) | 0.040703 | 0.191897 | 0.212106 | 0.837329 |
| pH | -1.01818 | 0.479743 | -2.12234 | 0.066582 |
| Urea concentration  (%) | -86.922 | 23.98714 | -3.62369 | 0.006746 |
| Wheat flour concentration (%) | 0.915807 | 0.239871 | 3.817908 | 0.005105 |
| K_2_HPO_4_ concentration (%) | -9.29906 | 4.797428 | -1.93834 | 0.088573 |
| MgSO_4_.7H_2_O concentration  (%) | 270.2059 | 95.94855 | 2.816154 | 0.022628 |
| ZnSO_4_ concentration (%) | -421.982 | 191.8971 | -2.199 | 0.059086 |

**Supplementary table (2): Analysis of Box-Behnken design constructed for optimizing the enzyme productivity**

| Source | SS^a^ | | DF^b^ | | MS^c^ | F-value | | *P*-value | |  |
| --- | --- | --- | --- | --- | --- | --- | --- | --- | --- | --- |
| Model | 222.24 | 9 | | 24.69 | | | 14.24 | | 0.0046 | |
| A-Urea concentration (%) | 13.97 | 1 | | 13.97 | | | 8.05 | | 0.0363 | |
| B-Wheat flour concentration (%) | 48.92 | 1 | | 48.92 | | | 28.21 | | 0.0032 | |
| C-MgSO_4_.7H_2_O concentration | 2.63 | 1 | | 2.63 | | | 1.52 | | 0.2729 | |
| AB | 2.6 | 1 | | 2.6 | | | 1.5 | | 0.2753 | |
| AC | 7.88 | 1 | | 7.88 | | | 4.54 | | 0.0862 | |
| BC | 62.78 | 1 | | 62.78 | | | 36.2 | | 0.0018 | |
| A^2^ | 44.57 | 1 | | 44.57 | | | 25.7 | | 0.0039 | |
| B^2^ | 0.1274 | 1 | | 0.1274 | | | 0.0735 | | 0.7972 | |
| C^2^ | 32.37 | 1 | | 32.37 | | | 18.66 | | 0.0076 | |
| Residual | 8.67 | 5 | | 1.73 | | |  | |  | |
| Lack of Fit | 8.17 | 3 | | 2.72 | | | 10.89 | | 0.0853 | |
| Pure Error | 0.5004 | 2 | | 0.2502 | | |  | |  | |
| Cor Total | 230.91 | 14 | |  | | |  | |  | |
| ^a^ Sum of squares  ^b^ Degrees of freedom  ^C^ Mean square | | | | | | | | | |  |

**Supplementary table (3): Analysis of Box-Behnken design**

| **Source** | | **SS^a^** | | **DF^b^** | **MS^c^** | **F-value** | ***P*-value** |
| --- | --- | --- | --- | --- | --- | --- | --- |
| Model | | 4206.45 | | 9 | 467.38 | 5.90 | 0.0144 |
| A- EWP concentration | | 235.45 | | 1 | 235.45 | 2.97 | 0.1284 |
| B- PEI concentration | | 298.8 | | 1 | 298.8 | 3.77 | 0.0933 |
| C- PEI pH | | 1606.93 | | 1 | 1606.93 | 20.28 | 0.0028 |
| AB | | 3.21 | | 1 | 3.21 | 0.04 | 0.8462 |
| AC | | 39.93 | | 1 | 39.93 | 0.50 | 0.5007 |
| BC | | 3.79 | | 1 | 3.79 | 0.05 | 0.833 |
| A² | | 1786.75 | | 1 | 1786.75 | 22.55 | 0.0021 |
| B² | | 113.98 | | 1 | 113.98 | 1.44 | 0.2694 |
| C² | | 25.79 | | 1 | 25.79 | 0.33 | 0.5861 |
| Residual | | 554.58 | | 7 | 79.23 |  |  |
| Lack of Fit | | 346.4 | | 3 | 115.47 | 2.22 | 0.2284 |
| Pure Error | | 208.18 | | 4 | 52.05 |  |  |
| Cor Total | | 4761.03 | | 16 |  |  |  |
| ^a^ Sum of squares  ^b^ Degrees of freedom  ^C^ Mean square | |  |  |  |  |  |  |
